# PCSK9 inhibitors in neurodegenerative disorders: mechanisms, therapeutic potential, and clinical implications

**DOI:** 10.1186/s40035-026-00568-y

**Published:** 2026-08-18

**Authors:** Jia Dong James Wang, Andrea York Tiang Teo, Bin Xiao, Yinxia Chao, Zhi Dong Zhou, Brendan Jen-Wei Tan, Ling-Ling Chan, Elvira Rosalie, Eng-King Tan

**Affiliations:** 1 Lee Kong Chian School of Medicine, Nanyang Technological University, Singapore, Singapore; 2 Department of Internal Medicine, National University Hospital, Singapore, Singapore; 3 Neuroscience and Behavioural Disorders, Duke-NUS Medical School, Singapore, Singapore; 4 Yong Loo Ling School of Medicine, National University of Singapore, Singapore, Singapore; 5 Department of Neuroradiology, Singapore General Hospital, National Neuroscience Institute, Singapore, Singapore; 6 Department of Neurology, Singapore General Hospital Campus, National Neuroscience Institute, Singapore, Singapore

**Keywords:** PCSK9 inhibitors, Neurodegenerative diseases, Alzheimer’s disease, Parkinson’s disease, Blood–brain barrier, Amyloid beta-peptides, Cholesterol metabolism, Neuroinflammation, Cognition disorders

## Abstract

Proprotein convertase subtilisin/kexin type 9 (PCSK9) plays a role in hepatic cholesterol metabolism via low density lipoprotein receptor degradation. PCSK9 inhibitors have revolutionized lipid-lowering therapy, providing robust reduction of cardiovascular risk. Large-scale randomized controlled trials and long-term extensions (e.g., FOURIER and EBBINGHAUS trials) have shown no significant adverse effects of PCSK9 inhibitors on neuropsychological testing or patient-reported cognitive outcomes, even with prolonged and intensive lowering of low-density lipoproteins. Pre-clinical studies also suggest PCSK9 as a key regulator of neurobiological processes, including synaptic plasticity, amyloid-beta clearance, neuroinflammation, and blood–brain barrier integrity. Human genetic studies revealed complex, sometimes conflicting associations between PCSK9 variants and risk of Alzheimer’s disease, Parkinson’s disease, vascular dementia, and amyotrophic lateral sclerosis. In this review, we highlight the pathophysiologic mechanisms, emerging experimental therapeutics and clinical implications of PCSK9 inhibition in neurodegenerative disorders. Long-term adequately powered trials with robust neuropsychological, biomarker, and imaging endpoints, as well as mechanistic studies in human-derived models are needed to establish the cardiovascular and neurocognitive implications of PCSK9 inhibition and guide precision medicine strategies for those at elevated risk of neurodegeneration.

## Background

Proprotein convertase subtilisin/kexin type 9 (PCSK9) is a serine protease best known for its role in cholesterol metabolism, emerging as a pivotal therapeutic target in cardiovascular disease. PCSK9 regulates cholesterol homeostasis primarily by targeting hepatic low-density lipoprotein receptors (LDLRs) for lysosomal degradation, which reduces LDLR recycling and elevates circulating cholesterol (LDL-C) levels [1]. Elevated LDL-C is a major risk factor for atherosclerosis, leading to an increased incidence of cardiovascular diseases such as myocardial infarction and stroke [2]. As such, PCSK9 is emerging as a crucial therapeutic target for lipid-lowering therapies [3]. The development of PCSK9 inhibitors, including monoclonal antibodies (e.g. alirocumab, evolocumab) and small interfering RNA (siRNA) agents (e.g. inclisiran), has revolutionized the management of hypercholesterolemia, offering robust LDL-C reduction and improved cardiovascular outcomes beyond traditional drugs such as statins [4, 5]. While the physiological consequences of PCSK9 activity are distinct in the periphery and the central nervous system (CNS), the underlying molecular mechanisms, primarily the binding and lysosomal degradation of LDLR family members, are largely conserved across both compartments.

Emerging evidence suggests that the role of PCSK9 extends beyond cholesterol metabolism, implicating it in inflammation, immune regulation, and neurodegenerative processes. Recent studies have revealed a potential connection between PCSK9 and neurodegenerative disorders such as Alzheimer’s disease (AD), Parkinson’s disease (PD), and vascular dementia, raising important questions about its contribution to neurodegenerative pathology [6, 7].

PCSK9 is expressed in key brain regions—including the cerebral cortex, hippocampus, and cerebellum—where it influences neuronal survival, synaptic plasticity, and neuroinflammatory responses [8]. Preclinical studies have identified PCSK9 expression in neurons, astrocytes, and microglia, demonstrating its role in modulating neuroinflammatory pathways, amyloid-beta (Aβ) clearance, and synaptic function [7]. Genome-wide association studies (GWAS) have further strengthened this link by identifying *PCSK9* polymorphisms associated with increased AD risk [9]. In addition, transcriptomic analyses have implicated dysregulated *PCSK9* expression in both AD and PD [10, 11].

Despite these findings, the mechanistic interplay between PCSK9 biology and neurodegeneration remains incompletely understood. For example, while PCSK9 has a well-established role in LDLR degradation thereby regulating cholesterol metabolism, emerging evidence suggests that it may also impair Aβ clearance through degradation of LDLR-related protein 1 (LRP1), a key receptor facilitating Aβ transport across the blood–brain barrier (BBB) [7]. The interactions of PCSK9 with apolipoprotein E (APOE) further suggest complexity in the modulation of amyloid pathology [12]. Moreover, PCSK9 exacerbates neuroinflammation by regulating cytokine release in microglia and may contribute to tau hyperphosphorylation through interactions with LRP1 [13].

Importantly, preclinical studies showed that PCSK9 inhibition may hold neuroprotective potential. Inhibition of PCSK9 reduces oxidative stress, mitigates neuroinflammation, and alleviates neuropathological changes in models of neurodegeneration [14]. In addition, PCSK9 inhibitors can diminish oxidative stress and modulate neuroimmune responses in the brain following chronic alcohol exposure [15]. Similarly, siRNA-mediated *PCSK9* silencing has demonstrated benefits in reducing neurovascular damage in murine models of stroke, highlighting possible cerebrovascular protective effects [13].

Given these mechanistic links between PCSK9, lipid metabolism, and neurodegenerative processes, there is growing interest in repurposing PCSK9 inhibitors as neuroprotective therapies. Here we draw attention to the pathophysiology of PCSK9 inhibitors, highlight current clinical associations and propose future directions for subsequent studies for the use of PCSK9 inhibitors for neurodegenerative disorders. Having outlined the compartmentalized functions of PCSK9 in systemic versus CNS contexts, we next examine its canonical role in lipid and cholesterol metabolism, which provides the foundation for understanding its broader neurobiological impact.

## PCSK9-mediated regulation of lipid and cholesterol homeostasis

Lipids, including cholesterol and triglycerides, are essential for cellular structure, energy metabolism, and signaling. Due to their hydrophobic nature, they are transported in the bloodstream, packaged into lipoprotein particles. These particles vary in density and composition, ranging from chylomicrons (triglyceride-rich) to very low-density lipoproteins (VLDL), intermediate-density lipoproteins, low-density lipoproteins (LDL), and high-density lipoproteins [2].

The LDLR is a cell surface protein that plays a central role in lipid clearance, particularly in the liver. LDLR binds to apolipoproteins on lipoprotein particles, primarily apolipoprotein B100 (ApoB100) on LDL and ApoE on VLDL and remnant particles, and mediates their internalization via clathrin-mediated endocytosis [1]. Once internalized, the lipoprotein particle is delivered to endosomes, where the acidic environment triggers dissociation of the ligand from the receptor. The lipoprotein cargo is directed to lysosomes for degradation and release of cholesterol, while LDLR is recycled back to the cell surface for subsequent rounds of lipoprotein uptake [16].

ApoE serves as a high-affinity ligand for LDLR and related receptors. Synthesized primarily by the liver and by astrocytes in the CNS, ApoE is a key component of VLDL, intermediate-density lipoproteins, and high-density lipoprotein particles [12]. In the periphery, ApoE facilitates the hepatic clearance of triglyceride-rich lipoprotein remnants, thereby contributing to systemic lipid homeostasis. In the CNS, where lipoproteins do not cross the BBB, ApoE is produced locally by astrocytes and mediates cholesterol transport to neurons via LDLR family members, including LDLR, LRP1, and apolipoprotein E receptor 2 (ApoER2) [17].

**Fig. 1:**
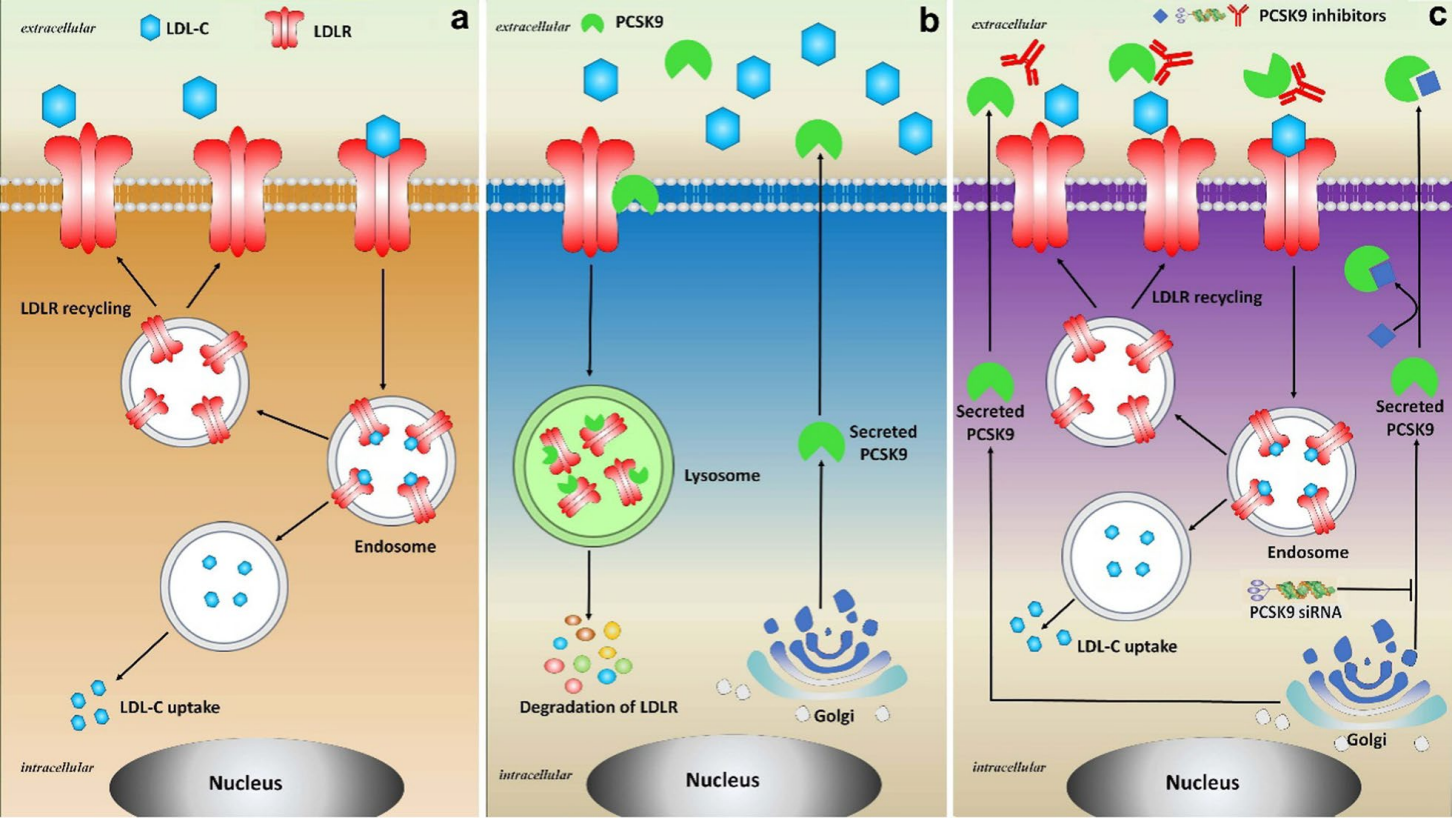
PCSK9 pathophysiology: pharmacokinetics and pharmacodynamics of PCSK9 inhibitors. **a** The LDLR is a transmembrane receptor primarily expressed in hepatocytes. Once the extracellular LDL-C binds to LDLR, the LDLR-LDL-C complex is internalized via clathrin-mediated endocytosis. In the acidic environment of endosomes, LDL-C dissociates from LDLR and is released for cellular use, while LDLR is recycled back to the cell surface, allowing it to bind and remove more LDL-C from circulation. **b** PCSK9 is a key regulator of LDLR recycling and cholesterol homeostasis, which can be synthesized inside cells and secreted outside of cells. PCSK9 binds to LDLR to form PCSK9-LDLR complex, which is then internalized and delivered to lysosomes for degradation. This process reduces LDLR availability on cell membrane, leading to impaired LDL-C uptake and increased circulating LDL-C levels. **c** PCSK9 inhibitors, such as small-molecule inhibitors, monoclonal antibodies and small interfering RNAs, effectively block PCSK9 activity. The small-molecule inhibitors directly inhibit PCSK9’s function inside the cell rather than targeting circulating PCSK9. The monoclonal antibodies bind to circulating PCSK9, preventing it from interacting with LDLR. The small interfering RNAs target PCSK9 mRNA in liver cells, reducing PCSK9 protein production. These therapies restore LDLR recycling, increasing hepatic LDL uptake and significantly lowering plasma LDL-C levels (C)

PCSK9 is a central regulator that modulates this recycling pathway. The canonical mechanism (Fig. 1) involves PCSK9 binding to the LDLR on the hepatocyte surface. This targets the entire complex for lysosomal degradation, effectively preventing the receptor from being recycled back to the cell membrane and thereby reducing hepatic clearance of circulating LDL-C. However, it remains uncertain whether the LDLR-mediated pathway could explain the role of PCSK9 in neurodegeneration, as other LDLR family members may contribute significantly to neuronal biology.

Therapeutic inhibition of PCSK9, achieved through monoclonal antibodies (e.g. evolocumab, alirocumab), siRNA therapies (e.g. inclisiran), or emerging gene-editing strategies, has been shown to enhance LDLR recycling and markedly lower plasma LDL-C levels [18–20]. Specifically, siRNA-based approaches such as inclisiran, silence *PCSK9* expression in hepatocytes, enabling sustained LDL-C reduction with two doses a year and offering clear advantages for long-term adherence [18].

Clinical trials, including FOURIER and ODYSSEY, have demonstrated that monoclonal antibodies targeting PCSK9 can achieve 50%–60% reductions of LDL-C, accompanied by a 15%–20% decrease in major adverse cardiovascular events [20]. Moreover, CRISPR-mediated gene-editing strategies aimed at knocking down *PCSK9* expression in the liver have shown promising results in preclinical models, with studies in *Macaca fascicularis* monkeys demonstrating sustained reductions of LDL-C levels by approximately 60% over several months [21].

Beyond its established role in LDL-C metabolism, PCSK9 has also been implicated in the regulation of triglyceride-rich lipoproteins, suggesting a broader influence on systemic lipid homeostasis. These findings underscore the therapeutic potential of PCSK9 inhibition not only for lowering LDL-C but also for addressing a wider spectrum of dyslipidaemia-related cardiovascular risk [22].

## Translational considerations: BBB penetration and indirect mechanisms

A fundamental question is whether the systemically administered PCSK9 inhibitors can reach the brain parenchyma. Monoclonal antibodies (evolocumab, alirocumab) are large molecules (~ 150 kDa) with limited BBB penetration under normal conditions [23]. siRNA agents (inclisiran) are even larger and do not cross the BBB. This suggests that any CNS effects observed in clinical trials are unlikely to result from direct PCSK9 inhibition within the brain parenchyma.

Then how might peripheral PCSK9 modulation influence CNS pathology? Several indirect mechanisms are plausible. First, PCSK9 inhibitors redue systemic LDL-C and atherosclerosis, thereby improving endothelial function and decreasing cerebrovascular disease burden, leading to secondary benefits on cognition by reducing white matter damage and silent infarcts. Second, PCSK9 inhibition decreases circulating pro-inflammatory cytokines such as interleukin (IL)−6 and tumor necrosis factor alpha (TNF-α)[24], and this peripheral immune modulation could attenuate neuroinflammation through reduced peripheral-immune crosstalk and microglial activation. Third, BBB dysfunction in disease states, such as hyperlipidemia, chronic inflammation, or neurodegeneration, compromises the barrier integrity [25–27], potentially allowing peripheral PCSK9 inhibitors, inflammatory mediators, or even small fractions of therapeutic antibodies to enter the CNS and directly influence pathological processes. Fourth, locally produced CNS PCSK9 by neurons and glia [8, 13] may itself be a relevant pathological target; however, whether systemically administered inhibitors could affect this pool, or whether effective central inhibition requires dedicated CNS-penetrant compounds, remains unknown. Rousselet et al. (2011) demonstrated that PCSK9 siRNA reduces neurovascular damage and improves neurobehavioral outcomes in a murine stroke model, providing direct evidence that inhibiting PCSK9, even locally, can confer neuroprotection [23]. Thus, while direct CNS target engagement by current PCSK9 inhibitors remains unproven, multiple indirect pathways could mediate the neurological effects. This distinction has important implications for interpreting clinical cognitive data and for designing neuroprotective trials in the future.

## Effects on local neuronal function: myelination and receptor-mediated signalling

Beyond its well-established role in systemic lipid metabolism, emerging evidence suggests that PCSK9 also exerts important effects on local neuronal function, particularly in regulating myelination, neuronal development, and synaptic integrity [13, 28, 29]. PCSK9 was initially identified in the brain, with expression levels peaking during embryonic development, a period characterized by intense neurogenesis [13]. Within the CNS, PCSK9 appears to regulate the abundance of LDLR and related receptors, thereby influencing cholesterol uptake by neurons and glial cells [28, 30].

### Myelination and cholesterol homeostasis

Cholesterol is an essential component of myelin [31]. Approximately 20% of myelin’s dry weight consists of cholesterol, underscoring its critical role in myelination [32]. Additionally, cholesterol plays essential roles in the structure and functions of neuronal membranes, particularly in the organization of lipid rafts. These specialized microdomains are vital for synaptic formation, neurotransmitter release, and the maintenance of synaptic plasticity [33]. Unlike peripheral tissues that rely on dietary intake and hepatic synthesis for cholesterol supply, the brain must produce its own cholesterol de novo by neurons, astrocytes, and oligodendrocytes, owing to the restrictive nature of the BBB. Crucially, while glial cells (astrocytes and oligodendrocytes) are robust cholesterol producers, adult neurons possess a limited capacity for autonomous cholesterol synthesis; therefore they rely on an "astrocyte-neuron shuttle" for cholesterol supply [34–36]. In this metabolic partnership, astrocytes synthesize cholesterol, package it into ApoE-containing lipoproteins, and secrete it for neuronal uptake via members of the LDLR family, including LDLR, LRP1, and ApoER2 [17].

PCSK9 regulation is particularly important for oligodendrocytes, the myelinating cells of the CNS, which depend on an adequate supply of cholesterol for proper function and myelin synthesis [37]. PCSK9 modulation of LDLR availability in the CNS may have direct consequences for neurodevelopment as well as for the progression of neurodegenerative diseases [7]. Specifically, PCSK9-mediated degradation of LDLR and other family members may limit cholesterol uptake in both neurons and oligodendrocytes, potentially exacerbating white matter damage, destabilizing synapses, and impairing remyelination [8, 29].

By effectively "starving" neurons from glial-derived cholesterol, elevated PCSK9 levels may compromise the structural integrity required for synaptic signaling and increase neuronal vulnerability to toxic insults [38]. Disruptions in PCSK9 activity have been associated with altered neuronal lipid composition, impaired myelination, and elevated neuroinflammatory responses [8, 29]. Yet, the extent to which the PCSK9-driven receptor degradation directly impairs remyelination in human disease remains unresolved, highlighting a key translational gap. A critical distinction must be made at this point: these direct effects of PCSK9 on CNS cells must be distinguished from its indirect effects on the brain through systemic mechanisms. By elevating circulating LDL-C, PCSK9 promotes atherosclerosis and cerebrovascular disease, which may lead to white matter damage, silent infarcts, and vascular cognitive impairment independent of its direct actions on neurons and glia. Understanding whether PCSK9 inhibition confers neuroprotection through direct CNS mechanisms, indirect vascular benefits, or both, remains a key translational question.

Experimental studies support these mechanistic links. Overexpression of PCSK9 in murine models reduces LDLR availability in neurons and glial cells, leading to diminished cholesterol uptake and compromised myelin maintenance [39]. Conversely, PCSK9 knockout mice exhibit increased brain LDLR levels, which may facilitate remyelination after injury [39]. These findings suggest that targeting PCSK9 may represent a novel therapeutic strategy to support remyelination in demyelinating disorders such as multiple sclerosis or to counteract age-related myelin degeneration [39].

### ApoER2 and VLDLR: mediators of synaptic plasticity

Beyond the role in cholesterol uptake, LDLR family members can directly regulate synaptic function. These receptors are highly expressed in neurons and glial cells, where they regulate synaptic function, dendritic spine morphology, and neuronal migration. ApoER2, in particular, is a key component of the Reelin signaling pathway, which governs synaptic plasticity and long-term potentiation [40, 41]. Reelin binding to ApoER2 activates intracellular cascades that modulate NMDA receptor function and protein kinase A-dependent stabilization of synaptic changes [41]. PCSK9-mediated degradation of ApoER2 and VLDLR disrupts this cascade, impairing LTP and linking cholesterol receptor turnover to cognitive outcomes [40].

In neurons, ApoER2 and VLDLR are concentrated at postsynaptic densities, where they transduce Reelin signals essential for dendritic spine maturation and maintenance [40]. This postsynaptic localization positions them to directly influence synaptic structure and function; their degradation by PCSK9 therefore has immediate consequences for synaptic integrity independent of the effects on cholesterol uptake. Structurally, effective LTP is associated with enlargement of dendritic spine heads and shortening of spine necks, changes that enhance electrical compartmentalization and synaptic efficacy [42]. PCSK9 activity has been implicated in the reduction of these mature, mushroom-shaped spines, leading to diminished synaptic density and functional connectivity.

Consistent with these mechanistic findings, inhibition of peripheral PCSK9 using monoclonal antibodies such as alirocumab in AD mouse models prevents the hippocampal-dependent memory impairment [43]. This functional rescue correlates with reduced Aβ pathology in the hippocampus and prefrontal cortex, strongly supporting the concept that the PCSK9-driven receptor turnover links synaptic dysfunction to cognitive decline and neurodegeneration.

### LRP1

LRP1 is essential for the clearance of Aβ across the BBB. At the BBB, LRP1 is highly expressed on the abluminal membrane of brain endothelial cells, where it internalizes Aβ from the interstitial fluid for efflux across the endothelium and into the circulation [25]. By facilitating efflux of soluble Aβ from the brain into the circulation, LRP1 protects against amyloid accumulation. Canuel et al. (2013) demonstrated that PCSK9 interacts with LRP1 and promotes its degradation, thereby reducing cell surface levels of this critical receptor [44]. This direct interaction provides the molecular basis for the effect of PCSK9 on LRP1-dependent processes in the CNS.

The functional consequences of this interaction for AD have been elucidated by Mazura et al. (2022), who demonstrated that PCSK9 reduces LRP1-mediated Aβ clearance across the BBB [43]. Critically, this study also provided evidence that pharmacological inhibition of PCSK9 (e.g., with alirocumab) increases peripheral and capillary LRP1 levels, thereby reducing cerebral Aβ burden and improving memory in preclinical models.

Beyond the BBB, LRP1 is also expressed in neurons, where it modulates tau phosphorylation through interactions with kinases including glycogen synthase kinase-3β (GSK-3β), and in microglia, where it regulates inflammatory signalling [45]. Thus, PCSK9-mediated LRP1 degradation may have cell type-specific consequences: impaired Aβ clearance at the endothelium, enhanced tau pathology in neurons, and dysregulated inflammation in microglia.

Together, these brain-specific targets highlight that the effects of PCSK9 in the CNS extend far beyond cholesterol metabolism. Through coordinated effects on LDLR (for myelination), ApoER2 and VLDLR (for synaptic plasticity), and LRP1 (for Aβ clearance and inflammatory signaling), PCSK9 influences multiple aspects of neuronal health. This receptor-specific regulation provides a mechanistic framework for understanding how PCSK9 inhibition may confer neuroprotective benefits—by preserving myelin integrity, maintaining synaptic function, enhancing protein clearance, and attenuating inflammation—beyond its established cardiovascular effects. These interconnected mechanisms will be explored further in the context of specific neurodegenerative diseases.

## Neuroinflammation

Having established the expression patterns and preclinical evidence for PCSK9 in the CNS, we next examine its role in neuroinflammation, a key driver of neurodegenerative pathology***.***

PCSK9 has recently been recognised as a critical modulator of neuroinflammation. It modulates microglial activity through critical targets including signalling receptor CD36, toll-like receptor (TLR), and nuclear factor kappa-B (NF-ĸB) activation. As the primary resident immune cells in the CNS, microglia are highly sensitive to changes in the immune environment. Their activation triggers an inflammatory cascade that exacerbates Aβ deposition, a hallmark of AD pathogenesis.

CD36, a multifunctional cell surface marker, is implicated in multiple pro-inflammatory signalling pathways and binds directly with Aβ fibrils, initiating downstream pathways[24]. Peripheral PCSK9 influences the CD36-mediated lipid uptake in macrophages, suppresses ABCA1-dependent cholesterol efflux, and amplifies systemic inflammation through cytokines such as IL-6 and TNF-α [24]. Microglial activation mediated by CD36–Aβ binding enhances secretion of inflammatory cytokines such as IL-1β, alongside activation of intracellular Tyr kinase-based transduction signals [8, 46]. This creates a feedback loop amplifying microglial activation, increasing Aβ aggregation and reactive oxygen species (ROS) production [45, 46]. While CD36 facilitates microglial Aβ clearance, upregulation of this receptor by PCSK9 shifts microglial function towards a pro-inflammatory state, promoting recruitment of adaptive immune responses, including T-cell activation [24, 39, 47]

In microglia, PCSK9 upregulation of CD36 shifts these cells toward a pro-inflammatory phenotype characterized by increased IL-1β and TNF-α production. In astrocytes, PCSK9-mediated LDLR degradation disrupts cholesterol export to neurons, potentially compounding the neuroinflammatory response. In oligodendrocytes, reduced LDLR availability impairs cholesterol uptake required for myelin synthesis and maintenance, potentially compromising white matter integrity[39].

PCSK9 also promotes oxidative stress through interconnected mechanisms that amplify neuronal injury. First, PCSK9-induced microglial activation generates ROS via NADPH oxidase (NOX) enzymes, particularly NOX2 [14]. Second, as cholesterol is essential for maintaining mitochondrial membrane fluidity and the organization of electron transport chain complexes, cholesterol depletion leads to mitochondrial depolarization, impaired adenosine triphosphate (ATP) production, and increased mitochondrial ROS generation [14, 45]. Therefore, by disrupting cholesterol homeostasis in neurons, PCSK9 compromises mitochondrial membrane integrity and function. Third, PCSK9-driven neuroinflammation and oxidative stress create a self-perpetuating feed-forward loop: ROS activates redox-sensitive transcription factors such as NF-κB, which further amplifies expression of pro-inflammatory genes, while the inflammatory cytokines impair mitochondrial function and antioxidant defences [48]. This vicious cycle is particularly detrimental in neurodegenerative diseases characterized by chronic, low-grade neuroinflammation. Recent studies demonstrate that PCSK9 inhibition attenuates oxidative stress in multiple preclinical models. In alcohol-exposed mice, PCSK9 inhibition significantly reduces neuronal ROS and lipid peroxidation, demonstrating that antioxidant effects are a direct consequence of PCSK9 blockade [15]. In stroke models, PCSK9 siRNA decreases oxidative damage and improves neurobehavioral outcomes [23]. In vitro, PCSK9 knockdown protects neurons from oxidant-induced injury [48].

Macrophages play a critical role in promoting or resolving inflammatory responses to injury [49]. Stimulatory molecules can facilitate the differentiation into specialised effectors that initiate and regulate immune responses [49]. Studies demonstrate that both LDL and PCSK9 drive local macrophage polarisation, promoting monocytic cell differentiation into a pro-inflammatory M1 phenotype, releasing IL-1, IL-6, TNF-α, and IL-1β [49, 50]. Elevated plasma cholesterol, oxidised LDL (oxLDL) and secreted cytokines further reinforce this polarisation, favouring the M1 inflammatory phenotype while suppressing M2 polarisation [49, 50].

Apart from direct immune modulation, PCSK9 may indirectly impair microglial function through disrupting the cholesterol balance. The resulting hypercholesterolemia increases BBB permeability and dysfunction, allowing excessive influx of inflammatory mediators into the CNS [45, 51–53]. Activated microglia perpetuate neurodegeneration through overproduction of inflammatory mediators, accelerating Aβ plaque formation and aggregation. Still, it is unclear whether the pro-inflammatory effects of PCSK9 are primary drivers of pathology or secondary to lipid dysregulation. Clarifying this is critical for therapeutic targeting.

Recent studies have shown that PCSK9 induces microglial activation via CD36 and TLR heterodimerisation, a process involving the TLR4/NF-ĸB signalling pathway. PCSK9 promotes neuroinflammation via TLR4/NF-κB and NLRP3 (NOD-like receptor pyrin domain-containing protein 3) inflammasome pathways in glial cells [7, 45, 53]. Tang et al. [54] reported that PCSK9 overexpression increases the oxLDL-mediated pro-inflammatory cytokine release, promoting TLR4 upregulation and subsequent NF-ĸB nuclear translocation [48]. Conversely, PCSK9 knockdown significantly attenuates the inflammatory effects [48], providing direct evidence for the anti-inflammatory potential of PCSK9 inhibition. Interestingly, the structural homology between resistin which binds TLR4, and the C-terminal cysteine-rich domain of PCSK9, suggests a shared mechanism of action [48, 53]. However, further mechanistic studies are required to clarify the role of PCSK9 in TLR4/NF-ĸB signalling [48].

Beyond the direct immune effects, inhibition of PCSK9 could also provide neuroprotective benefits by stabilizing cholesterol homeostasis and reducing neuroinflammation, although these possibilities require further investigation [13]. This neuroinflammatory environment, in turn, creates conditions conducive to protein misfolding and aggregation, which is a topic explored in the next section on proteinopathy.

## PCSK9 and proteinopathy: Aβ, tau, and α-synuclein

Beyond effects on cholesterol homeostasis and neuroinflammation, PCSK9 may also directly influence the metabolism and aggregation of pathogenic proteins characteristic of neurodegenerative diseases.

### Aβ

The most well-established link between PCSK9 and proteinopathy involves Aβ in AD. PCSK9-mediated degradation of LRP1 at the BBB directly impairs Aβ efflux from the brain parenchyma into the circulation. In 5 × FAD AD mice, PCSK9 knockout or pharmacologic inhibition with alirocumab restores LRP1 levels at the BBB, reduces cerebral Aβ plaque burden and improves cognitive performance [43]. Additionally, PCSK9 may influence Aβ production indirectly through effects on cholesterol homeostasis, as cholesterol-rich lipid rafts facilitate β-secretase activity and amyloidogenic processing of amyloid precursor protein [45].

### Tau

The relationship between PCSK9 and tau pathology remains less defined but mechanistically plausible. Several indirect pathways could link PCSK9 activity to tau hyperphosphorylation and aggregation. First, LRP1 directly interacts with tau and regulates its cellular uptake, secretion, and inter-neuronal spread [45]. PCSK9-driven degradation of LRP1 could therefore accelerate tau propagation along neuroanatomical pathways. Second, PCSK9-induced neuroinflammation activates stress kinases including GSK-3β and CDK5 (cyclin-dependent kinase 5), both of which phosphorylate tau at pathological sites [13]. Third, disruption of Reelin/ApoER2 signaling, resulting from PCSK9-mediated ApoER2 degradation, has been linked to tau pathology in experimental models, as Reelin deficiency exacerbates tau phosphorylation in tauopathy models [40]. While direct evidence linking PCSK9 to tau phosphorylation in humans is currently lacking, these converging mechanisms warrant investigation.

### α-Synuclein

**Fig. 2:**
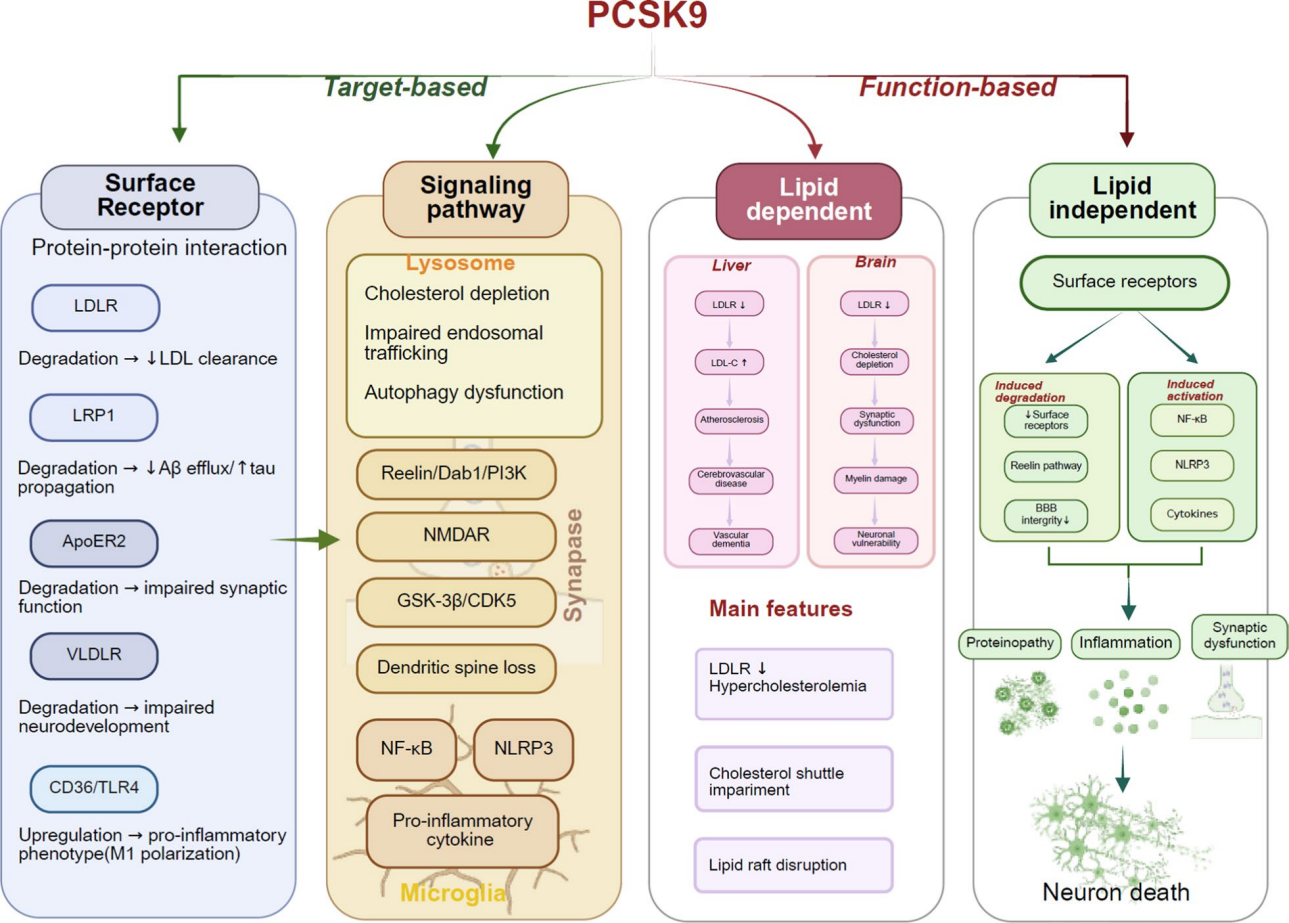
Comprehensive overview of PCSK9-mediated pathways in neurodegeneration, organized by mechanism of action. Left, target-based mechanisms. PCSK9 binds to surface receptors and targets them for degradation: LDLR (reducing LDL clearance), LRP1 (impairing Aβ efflux and promoting tau propagation), ApoER2 (disrupting synaptic function), VLDLR (impairing neurodevelopment), and CD36/TLR4 (upregulating pro-inflammatory M1 microglial polarization). Center-left, signaling pathways. Receptor degradation leads to downstream effects including lysosomal dysfunction (cholesterol depletion, impaired endosomal trafficking, autophagy impairment) and synaptic disruption (Reelin/Dab1/PI3K pathway inhibition, NMDAR dysfunction, GSK-3β/CDK5 activation, dendritic spine loss). Microglial activation occurs via NF-κB and NLRP3 inflammasome pathways, driving pro-inflammatory cytokine release. Center-right, lipid-dependent effects. In the liver, PCSK9 causes LDLR degradation, leading to elevated LDL-C, atherosclerosis, cerebrovascular disease, and vascular dementia. In the brain, PCSK9-mediated LDLR reduction results in cholesterol depletion, synaptic dysfunction, myelin damage, and increased neuronal vulnerability. Main features include hypercholesterolemia, impaired cholesterol shuttling, and lipid raft disruption. Right, lipid-independent effects. PCSK9 directly affects surface receptors by inducing degradation (reducing receptor presence, disrupting Reelin pathway, impairing BBB integrity) or activation (enhancing NF-κB, NLRP3, and cytokine signaling). These pathways collectively contribute to proteinopathy, neuroinflammation, synaptic dysfunction, and ultimately neuron death

The potential role of PCSK9 in α-synuclein pathology in PD represents a critical knowledge gap. Unlike Aβ, no direct receptor-mediated α-synuclein clearance pathway involving LDLR family members has been definitively established. However, α-synuclein aggregation is exquisitely sensitive to lipid membrane composition and cholesterol content [55]. By altering neuronal cholesterol homeostasis, particularly through disruption of the astrocyte–neuron cholesterol shuttle, PCSK9 could theoretically influence α-synuclein membrane binding, oligomerization, and aggregation. Cholesterol-rich lipid rafts facilitate α-synuclein clustering and fibril formation, and alterations in membrane cholesterol content modulate α-synuclein toxicity [55]. Additionally, PCSK9-driven oxidative stress and mitochondrial dysfunction create an environment conducive to α-synuclein misfolding and propagation. To date, no studies have directly examined effects of PCSK9 on α-synuclein in cellular or animal models of PD. Future experimental directions should prioritize evaluating PCSK9 inhibition in toxin-induced PD models (such as MPTP or 6-OHDA) and α-synuclein pre-formed fibril (PFF) models to determine if modulating lipid-membrane composition can attenuate dopaminergic loss and protein propagation (Fig. 2).

**Fig. 3:**
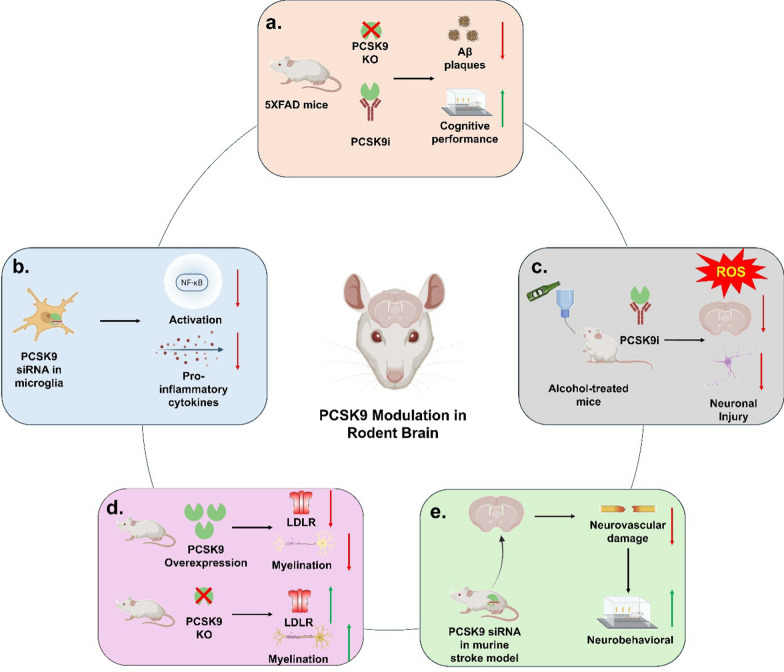
Schematic illustrating key findings from preclinical studies on the effects of PCSK9 inhibition or knockout on neurodegeneration in rodent models. **a** Mazura et al. (2022) showed that in 5 × FAD mice, PCSK9 knockout or pharmacologic inhibition reduces cerebral amyloid-beta (Aβ) plaque burden and improves cognitive performance via enhanced LRP1-mediated Aβ clearance [43]. **b** Tang et al. (2017) revealed that PCSK9 siRNA reduces NF-κB–mediated microglial activation and pro-inflammatory cytokine release, highlighting its anti-inflammatory potential [48]. **c** Wagner et al. (2024) demonstrated that in alcohol-treated mice, PCSK9 inhibition attenuates oxidative stress (ROS) and neuronal injury [15]. **d** In research by Jaafar et al. (2023), *PCSK9* knockdown enhances LDL receptor (LDLR) availability and promotes remyelination, whereas PCSK9 overexpression impairs myelination [39]. **e** Rousselet et al. (2011) showed that in a murine stroke model, PCSK9 siRNA reduces neurovascular damage and improves neurobehavioral outcomes [23]. Together, these models demonstrate the multifaceted neuroprotective effects of PCSK9 inhibition, including reduced neuroinflammation, oxidative injury, and Aβ pathology, and improved vascular and myelin integrity

In summary, PCSK9 appears to influence metabolism of pathogenic proteins through multiple convergent mechanisms: (i) directly affecting receptor-mediated clearance (Aβ via LRP1), (ii) indirectly through effects on neuroinflammation and kinase activation (tau), and (iii) affecting the lipid microenvironment that modulates protein aggregation (α-synuclein) (Fig. 3). These interconnected pathways highlight the potential for PCSK9 inhibition to exert broad effects across neurodegenerative diseases. However, targeted studies are needed to confirm these mechanisms in disease-specific contexts.

## Effects of PCSK9 inhibition on neurodegenerative disorders

### Effects of PCSK9 inhibitors on cognition and cognitive disorders: clinical evidence

PCSK9 inhibitors significantly decrease the LDL cholesterol levels beyond that typically achieved with statins alone, thus providing cardiovascular protection for patients who have not attained optimal lipid control with conventional therapies [56]. Early concerns arose that very low LDL or disruption of certain neuronal pathways might impair cognition, given the longstanding debate on statin-related cognitive symptoms [57, 58]. In response, regulatory agencies, including the Food and Drug Administration (FDA), request major PCSK9 inhibitor trials to systematically monitor neurocognitive events [59]. A robust body of clinical evidence strongly indicates that PCSK9 inhibition is unlikely to produce clinically meaningful disruption of cognition over time, although recent data raised the possibility of nuanced effects in specific domains or genotypes. Here we highlight various studies that have captured cognitive outcomes, synthesize findings across landmark trials, and explore *APOE* genotype as a potential moderator of risk or benefit with PCSK9 therapy. There are ongoing efforts to evaluate whether PCSK9 inhibitors might, in certain settings, confer subtle neuroprotective benefits, though caution is warranted when interpreting these emerging signals. A summary of the main findings of published clinical trials are summarised in Table 1.

**Table 1:** Summary of key studies examining cognition with PCSK9 inhibitors

Study & Year	Type of study/Population	Median age (years)	Cognitive/Reporting tests	PCSK9 inhibitors	Main findings
Giugliano et al. (2017) – EBBINGHAUS [60]	• Phase 3 RCT—FOURIER substudy • *n* = 1204 • Median follow-up: 19 months • Europe (~ 67%), North America (~ 26%), Asia–Pacific & South Africa (~ 7%)	~ 63	• Cambridge Neuropsychological Test Automated Battery (CANTAB): Executive function, Working memory, Episodic memory, Psychomotor speed	Evolocumab	• No significant cognitive differences between evolocumab and placebo • LDL levels (very low vs. moderate) did not correlate with cognitive scores
Zimmerman et al. (2025)—FOURIER Extension [61]	• Long-term open-label extension of EBBINGHAUS • *n* = 473 • Median follow-up: 5.1 years (max 7.2 years) • Europe and North America	~ 62	• Annual CANTAB testing: Spatial working memory strategy index	Evolocumab	• No significant executive function changes over time • LDL levels remained low without cognitive impairment
Gencer et al. (2020)—FOURIER Cognition [62]	• Secondary analysis from entire FOURIER trial • *n* = 22,655 • Median follow-up: 2.2 years • Europe (~ 63%), North America (~ 16%), Asia–Pacific & South Africa (~ 15%), Latin America (~ 6%)	~ 62	• Patient-reported Everyday Cognition (ECog) scale	Evolocumab	• Similar rates of subjective cognitive decline (~ 3.6%–3.7%) • Very low LDL cholesterol (< 20 mg/dL) not linked to perceived cognitive decline
Seijas-Amigo et al. (2023) – MEMOGAL [63]	• Prospective observational study • 12 Spanish hospitals: *n* = 158 • Median follow-up: 99 weeks • Northwest of Spain	60.6	• Montreal Cognitive Assessment (MoCA): Executive function, Language, Attention, Recall	Evolocumab and Alirocumab	• No major changes in global MoCA scores • Significant improvement in delayed recall memory • No difference between evolocumab and alirocumab
Janik et al. (2021) [64]	• RCT on High cardiovascular risk patients • *n* = 2176 (2086 were evaluated) • 96-week follow-up • White 81.7%, Black or African American 7.5%, Asian 2.2%, American Indian/Alaska Native 1.2%, Other 7.4%; Hispanic/Latino 12.9%	~ 63	• CANTAB: Spatial Working Memory Strategy, Paired Associate Learning, Reaction Time	Alirocumab	• No significant cognitive impairment • Small trends favoring PCSK9 inhibitors in reaction time and global composite scores
Korthauer et al. (2022)—APOE & PCSK9 inhibitors [65]	• Genetic substudy in FOURIER/EBBINGHAUS—*n* = 13,481 (28% ε4 carriers) • North America, Europe, Latin America, and Asia–Pacific	~ 63	• Everyday Cognition (ECog) scale • CANTAB (subset of 835)	Evolocumab	• No significant genotype-by-treatment interaction • *APOE* ε4 carriers had more memory complaints in placebo group but not in evolocumab group

#### Types of studies and endpoints for cognition

Studies using PCSK9 inhibitors have broadly evaluated four main categories of cognitive outcomes: (1) formal neuropsychological test performance, (2) patient-reported cognitive symptoms, (3) clinically diagnosed cognitive adverse events, and (4) long-term cognition (Table 1).

Randomized controlled trials and observational cohorts typically use validated test batteries like the Cambridge Neuropsychological Test Automated Battery (CANTAB) or Montreal Cognitive Assessment (MoCA) to quantify domains (e.g. executive function, working memory, psychomotor speed), as well as patient-centered questionnaires (e.g. the Everyday Cognition [ECog] survey) to capture subjective cognitive complaints [62–64].

##### Formal neuropsychological test performance

Two pivotal randomized trials employed objective cognitive testing to assess the impact of PCSK9 inhibition on neurocognitive function. The EBBINGHAUS trial enrolled 1204 patients in the FOURIER trial and followed them for a median of 19 months using the CANTAB [60]. The primary endpoint was the change in spatial working memory strategy (SWMS) index (lower scores indicate better performance). During the follow-up, SWMS scores changed by − 0.21 ± 2.62 in the evolocumab group versus − 0.29 ± 2.81 in the placebo group (*P* < 0.001 for noninferiority; *P* = 0.85 for superiority), indicating no significant detrimental effect [60]. Additional domains, including working memory errors, episodic memory errors, and psychomotor speed, were similarly neutral (all *P* > 0.05). Exploratory analyses revealed no link between achieving very low LDL (< 25 mg/dL) and cognitive decline.

A complementary alirocumab trial then randomized 2176 high-risk patients to receive alirocumab or placebo over 96 weeks (of whom 2086 had CANTAB cognitive domain Spatial Working Memory Strategy (SWMS) assessments) [64]. The primary outcome based on the CANTAB SWMS z-score, showed near-identical changes after two years: − 0.180 (alirocumab) vs. − 0.200 (placebo), yielding a treatment difference of − 0.020 (95% CI: − 0.094 to 0.055; *P* = 0.6055) [64]. Secondary measures (paired associate learning, reaction time, global composite scores) were likewise comparable between groups, reinforcing a neutral effect of PCSK9 inhibition on formal cognitive testing.

##### Patient-reported cognitive symptoms

In the broader FOURIER trial (*n* = 27,564), 22,655 participants completed a self-reported end-of-study ECog questionnaire [62]. Over a median 2.2-year follow-up, 3.7% of the evolocumab arm versus 3.6% of the placebo group reported cognitive decline (*P* = 0.62). Memory subscores (≈6%) and executive-function subscores (≈3.6%–3.7%) did not differ between arms. Notably, those achieving LDL < 20 mg/dL were no more likely to report decline compared to participants with LDL ≥ 100 mg/dL (3.8% vs. 4.5%, *P* = 0.57), suggesting that even very low LDL levels did not prompt subjective memory or executive dysfunction [62]. A summary of the cognitive adverse effects is provided in Table 2.

**Table 2:** Neurocognitive adverse events in PCSK9 inhibitor trials

Study	Study population/type of cohort	Age	PCSK9 inhibitor	Sample size	Neurocognitive findings
ODYSSEY CHOICE I [66]	Multinational trial population from North America and Europe (with sites in 8 countries) Hypercholesterolaemia at moderate–very-high cardiovascular risk; on maximally tolerated statin or no statin (including statin-intolerant subgroup)	Mean: 59–62 years	Alirocumab	803	No significant difference in neurocognitive events
FOURIER (2017)[4]	Predominantly White patients from Europe and North America, with additional representation from Asia, Africa, and Latin America Established atherosclerotic cardiovascular disease (prior myocardial infarction/stroke/peripheral artery disease) on statin background therapy	Median: 62–63 years	Evolocumab	27,564	No difference in neurocognitive adverse events (~ 1.5% in both groups)
MENDEL-2 [67]	International trial in statin-naïve patients (primarily conducted in North America and Europe) Statin-naïve primary hypercholesterolaemia	Mean: 53 years	Evolocumab	614	No significant difference in neurocognitive events
ODYSSEY LONG TERM [68]	Large global trial population across 27 countries (predominantly Western countries) High cardiovascular risk hypercholesterolaemia (including familial hypercholesterolaemia) despite maximally tolerated statins	Mean: 60.4–60.6 years	Alirocumab	2341	Higher neurocognitive events in alirocumab (1.2%) vs. placebo (0.5%), but not statistically significant (*P* = 0.17)
OSLER [69]	Pooled multinational cohort from prior trials (North America, Europe, and other regions) Mixed hypercholesterolaemia phenotypes (including FH/non-FH; secondary prevention subsets)	Mean: 57.8–58.2 years	Evolocumab	4465	Slightly more neurocognitive events in evolocumab group, but no statistical significance reported
FOURIER-OLE [70]	Long-term extension with patients primarily from Europe (East & West) and the United States Open-label extension of FOURIER (same ASCVD phenotype; longer follow-up)	Mean: 62.4 years	Evolocumab	6635	No significant difference in neurocognitive event incidence
ODYSSEY OUTCOMES [71]	Global post-ACS cohort from 49 countries across North/South America, Europe, Asia, Africa, etc Post–acute coronary syndrome (recent ACS) population on intensive statin therapy	Median: 59 years	Alirocumab	18,924	No significant difference in neurocognitive events (alirocumab vs. placebo: 1.5% vs. 1.8%)
ODYSSEY COMBO II [72]	Multinational trial spanning Europe, North America, Middle East (Israel), Africa, and Asia High cardiovascular risk hypercholesterolaemia despite maximally tolerated statin	Mean: 61.3–61.7 years	Alirocumab	720	No excess neurocognitive events reported
ODYSSEY ALTERNATIVE [73]	Statin-intolerant patients from an 8-country mix (Europe, North America, Middle East)	Mean: 62.8–64.1 years	Alirocumab	361	No significant difference in neurocognitive events
REGN727/SAR236553 Phase II [74]	HeFH patients from North American lipid clinics (U.S. and Canada) on intensive statin therapy	Mean: 51–56 years	Alirocumab	77	9 patients had nervous system disorders in the treatment arm
ODYSSEY Japan (Post-hoc) [75]	Exclusively Japanese patient population (familial and non-familial hypercholesterolemia on statin therapy)	Mean: 60–62 years	Alirocumab	216	No clear cognitive safety concerns
DESCARTES Trial [76]	Predominantly White, with smaller proportions of Black, Asian, and other racial groups in a multi-country cohort recruited from 88 centers in nine countries Hypercholesterolaemia across background therapies (diet alone vs statin/ezetimibe)	Mean: 51–58 years	Evolocumab	901	No significant neurocognitive events observed

##### Clinically diagnosed cognitive adverse events

Most studies, however, only monitored for investigator-reported adverse events such as confusion, amnesia, or clinically significant memory impairment. The overall incidence of such events remained low and was similar between the PCSK9 inhibitor and placebo groups. Early reports from non-blinded extension studies (e.g. OSLER) hinted at minor imbalances, but subsequent large-scale, randomized data did not confirm higher rates of clinically diagnosed cognitive deficits (Table 2) [69, 77, 78]. Real-world surveillance, such as the MEMOGAL registry, indicates a low frequency of these events across evolocumab and alirocumab recipients [63].

##### Long-term cognitive disorders or dementia risk

Two additional long-term investigations illustrate neutral or potentially favorable outcomes over longer observation periods. The MEMOGAL study assessed 158 patients with PCSK9 inhibitors (52% evolocumab, 48% alirocumab) over a median 99 weeks [63]. The total MoCA scores changed by + 0.28 points (95% CI − 0.17 to 0.73; *P* = 0.216), while delayed recall memory was improved significantly (+ 0.44; *P* = 0.001) [63]. The degree of LDL reduction did not predict these changes, and both PCSK9 inhibitors performed similarly, implying a neutral or mildly positive effect on memory-specific domains.

In the open-label EBBINGHAUS extension trial, 473 patients continued evolocumab therapy for a median 5.1 years (up to 7.2 years) [61]. Executive function (SWMS) scores remained stable in individuals originally randomized to evolocumab (mean change 0.1 ± 2.8; *P* = 0.49) or switched over from placebo (− 0.1 ± 2.5; *P* = 0.64), despite persistently low LDL levels (median ~ 35 mg/dL) [61]. At final assessment, scores were nearly identical in the two arms, underscoring the absence of progressive cognitive decline even under extended, intensive LDL-lowering for long periods.

Taken together, these findings suggest that PCSK9 inhibitors do not adversely affect cognition, whether assessed by standardized tests, subjective measures, or clinical diagnoses, and that no apparent long-term cognitive impairment risk emerges over prolonged follow-up. On the contrary, slight improvements in some cognitive subdomains (e.g. delayed recall) may occur. However, whether these reflect a genuine pharmacologic benefit or practice effects warrants further investigation. The key evidence regarding cardiovascular and neurological implications of PCSK9 inhibition is provided in Table 3.

**Table 3:** Comparative evidence for PCSK9 inhibitors in cardiovascular and neurological contexts

Domain	Cardiovascular evidence	Neurological evidence
Primary outcome trials	Robust reduction in major adverse cardiovascular (MACE) events (FOURIER, ODYSSEY Outcomes: 15%–20% risk reduction)	No trials with neurodegenerative disease progression as primary endpoint
Cognitive effects	Cognitive safety established; no increase in neurocognitive adverse events in > 50,000 patient-years of follow-up	Domain-specific improvements (delayed recall memory in MEMOGAL); no objective cognitive decline
Potential benefits	• LDL-C reduction (50%–60%) • Reduced atherosclerosis • Decreased MACE	• Enhanced Aβ clearance via LRP1 restoration • Reduced neuroinflammation • Attenuated oxidative stress • Preserved synaptic integrity
Key limitations	Long-term safety beyond 5 years still under investigation	• No trials in MCI/dementia populations • Limited preclinical data in PD, tauopathy, ALS models • BBB penetration of current inhibitors uncertain
Genetic evidence	PCSK9 loss-of-function variants associated with lower LDL-C and reduced CVD risk	Conflicting MR results: some studies suggest increased AD risk, others show neutral or protective effects
APOE interaction	*APOE* genotype influences lipid-lowering response	*APOE4* carriers show elevated CSF PCSK9 levels; may derive differential cognitive benefit
Mechanism of action	Direct: Enhanced hepatic LDLR recycling → increased LDL-C clearance	Indirect: Vascular benefits, reduced systemic inflammation; Direct (proposed): CNS receptor modulation (LRP1, ApoER2)

CVD, cardiovascular diseases

## *APOE* genotype, PCSK9 inhibitors, and long-term cognitive outcomes

APOE is central to lipid transport in both systemic and cerebral contexts, and the ε4 allele is strongly associated with AD risk. It has been hypothesized that the cholesterol-lowering therapies may have distinct effects among ε4 carriers. Korthauer et al. analyzed 13,481 FOURIER participants with *APOE* genotyping (28% ε4 carriers) [79]. In the placebo arm, the ε4/ε4 carriers showed higher odds of patient-reported memory decline on the ECog scale (*P* = 0.003 for trend; odds ratio = 1.46, 95% CI [1.03, 2.08]) compared with non-carriers. In contrast, there was no such relationship in the evolocumab arm (*P* = 0.50, OR = 1.18, 95% CI [0.83, 1.66]). Although the genotype-by-treatment interaction was not significant (*P* = 0.30), it may suggest that PCSK9 inhibition could mitigate the *APOE* ε4–related memory decline observed in placebo recipients [65]. Objective CANTAB testing in a subset of 835 patients, however, indicated no APOE-related modification of the relationship between treatment arm and CANTAB performance (*P* > 0.05). Some preclinical work has suggested that PCSK9 inhibition may favorably modulate amyloid pathways, potentially lowering dementia risk. However, these findings are preliminary and therefore require more targeted, long-term investigation.

Notably, recent studies have reported increased PCSK9 levels in both cerebrospinal fluid (CSF) and brain tissues of AD patients carrying the *APOE* ε4 allele [79, 80]. This genotype-specific elevation suggests that APOE4 may interact with PCSK9 to exacerbate amyloid pathology and neurodegeneration, potentially leading to higher AD risk in this population. These findings raise the intriguing possibility that PCSK9 inhibition may have differential effects based on *APOE* status, a hypothesis that warrants prospective evaluation in genotype-stratified clinical trials.

Given these findings, it is conceivable that PCSK9 inhibitors might reduce or neutralize certain *APOE* ε4-associated risks over time and potentially mitigate long-term cognitive disorders like Alzheimer’s dementia. Nonetheless, such a hypothesis must be viewed with caution, as current datasets lack the power to detect rare or subtle outcomes like progression to mild cognitive impairment or AD. Larger, longer studies with comprehensive neuroimaging or biomarker assessments are needed to clarify whether PCSK9 inhibitors could confer protective or disease-modifying effects for dementia. Building on this evidence of cognitive safety and potential domain-specific benefits, we will next examine the implications of PCSK9 inhibition for specific neurodegenerative diseases, beginning with AD.

## PCSK9 inhibitors and AD

AD is widely known to be associated with the dysregulation of brain cholesterol trafficking. Mechanistically, dysregulated cholesterol metabolism, mediated in part by PCSK9, contributes to Aβ accumulation and tau pathology [45]. Despite the above studies which indicate that PCSK9 inhibition may reduce the risk of AD, current clinical data remain controversial. Several studies found that PCSK9 levels in the CSF [81] and frontal cortices [82] were higher in AD individuals than in those without AD. However, a subsequent study showed that there was no difference in the CSF levels of PCSK9 between AD and non-AD subjects, despite a general increase of CSF PCSK9 concentration in patients with neurodegenerative disorders (not specifically in AD)[83]. A Mendelian randomisation study further suggested that genetically proxied PCSK9 inhibition was associated with a higher predicted risk of AD [84]. Further investigations of the clinical impact of PCSK9 inhibitors on AD are needed. The above human data, combined with the largely beneficial effects in preclinical models, indicate that PCSK9 inhibition is at worst neutral and potentially protective for AD, rather than harmful. Major gaps remain in reconciling the mechanistic complexity, such as on understanding how PCSK9 influences cholesterol and Aβ metabolism in the human brain. The potential benefit of PCSK9 inhibition in AD may therefore arise from both direct CNS effects (restoring LRP1-mediated Aβ clearance at the BBB) and indirect vascular benefits (reducing cerebrovascular disease burden that exacerbates cognitive decline). Furthermore, no clinical trials have tested PCSK9 inhibitors in AD progression.

In summary, current findings suggest that PCSK9 inhibition could be protective in AD by (1) restoring LRP1-mediated Aβ clearance across the BBB, (2) reducing microglial neuroinflammation and oxidative stress, and (3) improving cerebrovascular health through systemic lipid lowering. These mechanisms likely operate synergistically. For example, reduced vascular inflammation may further enhance LRP1 function, while decreased Aβ burden may attenuate microglial activation, suggesting that PCSK9 inhibition could modify multiple disease pathways simultaneously. Further studies (especially in human subjects and *APOE* ε4 carriers) are needed to clarify long-term cognitive outcomes.

## PCSK9 inhibitors and vascular dementia

Vascular dementia is a common cause of age-related dementia. The pathophysiology of vascular dementia varies, encompassing multiple subtypes, each with different degrees of tissue loss and impact on neural pathways [85, 86]. Given the close relationship between ischemic events and vascular dementia, management of vascular dementia commonly encompasses the treatment of comorbidities such as hypertension and hypercholesterolemia. Statins, for instance, are used for prevention of secondary strokes, and PCSK9 inhibitors are now used to achieve even tighter LDL control in patients with cerebrovascular disease. Recent studies on the association between serum cholesterol and the risk of vascular dementia point toward tighter cholesterol control as being beneficial in vascular dementia, though controversy remains on the effects of tight regulation of cholesterol on neurocognitive function [87, 88]. With regard to PCSK9 inhibition, one Mendelian randomisation study found no evidence that genetically lowered LDL cholesterol through PCSK9 variants decreased the risk of vascular dementia [89]. Another analysis showed that lifelong exposure to low PCSK9 levels and cumulative exposure to lower levels of LDL are not associated with adverse neurocognitive effects [90]. However, it must be noted that no trials to date have specifically evaluated PCSK9 inhibitors in patients with established vascular dementia or mild vascular cognitive impairment. Thus, direct evidence of cognitive improvement is lacking. The current consensus is that PCSK9 inhibition is at least cognitively safe in the context of vascular dementia, and it could be protective by lowering stroke recurrence. While the primary benefit in vascular dementia is likely indirect through reduced cerebrovascular events, direct effects on endothelial LRP1 and BBB integrity may also contribute, by synergistically preserving white matter integrity and cognitive function. Some even propose aggressive LDL lowering as a strategy to mitigate dementia risk [91]. In conclusion, for vascular dementia, PCSK9 inhibitors are likely neutral to beneficial: they strongly address vascular risk factors without measurable cognitive harm, and they may improve neurovascular health. The recent PISTIAS-2 trial [92], which incorporates cognitive and volumetric imaging endpoints in patients with intracranial atherosclerosis, will provide valuable data on whether intensive LDL lowering with PCSK9 inhibition protects against vascular cognitive impairment through combined vascular and neurovascular mechanisms.

## PCSK9 inhibitors and PD

The role of cholesterol homeostasis in PD progression has been discussed and debated [55]. High total serum cholesterol may result in slow progression of PD, and high plasma LDL levels have been associated with better motor performance in PD patients [93]. In another study, however, low LDL levels due to *PCSK9* genetic variants did not appear to increase the risk of PD [89]. Evidence on the effects of PCSK9 inhibition in PD has not been consistent as well. One case report suggested clinical improvement in a PD patient after receiving PCSK9 inhibition [94]. Yet, another analysis showed that PCSK9 inhibition provided no neuroprotective benefit in PD [84], while a separate study suggested that PCSK9 inhibitors increased the risk of PD (OR = 1.417, 95% CI = 1.178 to 1.657, *P* = 0.004) [95]. Overall, the literature appears to point towards a possible increased risk of PD with PCSK9 inhibition. This may potentially be due to the suppression of plasma LDL levels. No large trials have specifically assessed neurodegenerative outcomes in patients on PCSK9 inhibitors. Further investigations with long-term patient registries and neuroimaging approaches could help clarify whether PCSK9 inhibitors subtly influence PD onset or progression. Better-powered studies are needed to confirm whether lowering PCSK9 (and LDL-C) is truly detrimental in PD or if confounders (like reverse causation, wherein prodromal PD leads to low lipids) could explain the associations. Whether PCSK9 influences PD through direct effects on neuronal cholesterol homeostasis, indirect effects via systemic lipid alterations, or both, remains to be determined.

While the direct links between PCSK9 and α-synuclein remain unexplored, the convergence of cholesterol dyshomeostasis, oxidative stress, and neuroinflammation, all modulated by PCSK9 activity, represents a plausible pathway by which PCSK9 influences PD pathogenesis. The oxidative stress and mitochondrial dysfunction resulting from PCSK9-driven cholesterol dysregulation may be particularly relevant to dopaminergic neurons, which are exquisitely sensitive to oxidative damage [14]. Future studies should examine whether PCSK9 inhibition affects α-synuclein aggregation, dopaminergic neuron survival, or motor outcomes in preclinical PD models.

## PCSK9 inhibitors and epilepsy

The correlation between higher circulating lipid levels and an increased risk of epilepsy has been explored in several studies [96, 97]. The use of statins, for instance, has been suggested to reduce the incidence of post-stroke seizures and epilepsy [98–100] via its effects on nitric oxide metabolism. The effect of PCKS9 inhibitors on epilepsy, however, is less well-understood. In one study, the risk of juvenile absence epilepsy was associated with higher expression of PCSK9 (OR = 1.06, 95% CI: 1.02 to 1.09, *P* = 0.002) [101]. Another Mendelian randomization analysis showed that PCSK9 inhibitors were associated with a equivocal risk of epilepsy (OR, 1.112, 95% CI, 0.215–5.761, *P* = 0.902, random-effect model; OR, 0.693, 95% CI, 0.463–1.039, *P* = 0.076, fixed-effect model) [102]. Future studies on the expression of PCSK9 in epileptic brain tissues and retrospective analyses of seizure incidence in patients on PCSK9 inhibitors, would provide insights into the correlations. Until more definitive evidence emerges, PCSK9 inhibition appears to be either potentially protective or, at minimum, neutral with respect to epilepsy risk, consistent with the broader concept that improved vascular and metabolic health may contribute to neurological stability.

## PCSK9 inhibitors and amyotrophic lateral sclerosis (ALS)

Disorders of lipid metabolism have been widely reported in patients with ALS [103]. Though the role of lipid dysregulation in the progression of ALS is not fully elucidated, it is believed that the accumulation of cholesteryl esters and ceramides mediates oxidative stress in the motor neurons of ALS patients. Furthermore, high LDL levels and the *APOB* gene are significantly associated with an increased risk of ALS [104, 105]. Therefore, the use of PCSK9 inhibitors to modulate the disease progression of ALS has gained attention in recent years. In one study, inhibition of PCSK9 was not linked to ALS progression (*OR* = 0.95, 95% CI = 0.83–1.11; *P* = 0.51) [106]. Similarly, the expression of the *PCSK9* gene did not show significant association with the risk of ALS (OR = 1.10, 95% CI = 0.97–1.24; *P* = 0.143) [107]. In contrast, Huang et al. demonstrated a marginal but significant protective effect of PCSK9 inhibitors against ALS (OR = 0.89, 95% CI = 0.77–1.00, *P* = 0.048) [95]. Overall, current evidence does not indicate that PCSK9 inhibition worsens ALS outcomes, and some analyses suggest a possible protective association. This is an important observation given longstanding concerns that aggressive cholesterol lowering might be detrimental in neurodegenerative disorders. Future research could focus on whether subgroups of ALS (e.g., those with metabolic syndrome or certain genetic backgrounds) benefit from lipid-lowering interventions. Current evidence suggests that the use of PCSK9 inhibitors for cardiovascular indications in ALS patients is not contraindicated by neurodegenerative considerations.

Although randomized cardiovascular outcome trials and cognitive post-hoc studies showed that PCSK9 inhibitors do not cause short-term, clinically meaningful cognitive impairment, the neurological evidence base remains indirect. To date, most human data have been derived from cardiovascular cohorts in which neurodegenerative disease outcomes, CNS target engagement, and disease-relevant biomarkers were not primary endpoints. As a result, mechanistic inference and any putative neuroprotective implications remain largely extrapolated from preclinical work and genetic epidemiology.

## Translational implications for neurodegenerative diseases

The mechanistic framework outlined above suggests that PCSK9 inhibition could theoretically influence multiple neurodegenerative disease pathways through both direct and indirect mechanisms. However, the translational relevance varies considerably across disorders.

In AD, PCSK9 inhibition may confer benefit through direct effects on LRP1-mediated Aβ clearance at the BBB and indirect vascular benefits that reduce cognitive decline. Clinical data remain conflicting, with some studies showing elevated PCSK9 in AD brains [79, 81] while others reporting no difference [83]. Genetic studies also yielded inconsistent results [84, 89]. *APOE4* carriers may represent a particularly relevant subgroup given their elevated PCSK9 levels [79, 81].

For vascular dementia, the primary translational pathway is indirect—reducing cerebrovascular events and white matter damage through aggressive LDL-C lowering. Direct effects on endothelial LRP1 and BBB integrity may also contribute, though this requires confirmation.

In PD, the translational rationale is less clear. While observational studies have linked cholesterol to PD progression [55, 93], genetic data suggest that PCSK9 inhibition might paradoxically increase PD risk [95]. Whether this reflects direct neuronal effects or confounding effects remains unresolved.

In ALS and epilepsy, preliminary genetic studies suggest possible protective effects of PCSK9 inhibition [95, 101, 102], but mechanistic understanding is limited.

Critically, no clinical trial to date has been designed with the use of neurodegenerative disease progression as a primary endpoint. Thus, while the mechanistic framework supports further investigation, translational application of PCSK9 inhibitors to neurodegeneration remains to be hypothesis-generating rather than evidence-based. Dedicated trials incorporating disease-specific biomarkers, neuroimaging, and cognitive endpoints are urgently needed.

## Limitations of current studies

Despite encouraging evidence supporting the cardiovascular safety and the potential neuroprotective effects of PCSK9 inhibitors in pre-clinical studies, there are still gaps from molecular mechanisms to clinical outcomes, necessitating caution in interpretation and underscoring the need for further research in view of extrapolated mechanisms and limited clinical evidence.

### Limited clinical generalisability and inadequate follow-up

One of the pressing limitations is that current clinical trial data on cognitive safety largely derived from populations at relatively low risk of neurodegeneration. Older adults, *APOE* ε4 carriers, and patients with mild cognitive impairment or dementia are often excluded, leaving critical subgroups underrepresented. Moreover, the follow-up periods in most trials were short, typically one to two years, which may be insufficient to capture subtle or cumulative neurocognitive effects that would emerge only after decades of exposure. Neuropsychological assessments have also varied widely across studies, with many relying on screening tools or subjective questionnaires that may not detect domain-specific or early-stage changes.

### Translational gaps between models and humans

Preclinical models have provided valuable mechanistic insights into the role of PCSK9 in synaptic plasticity, Aβ clearance, neuroinflammation, and BBB integrity. However, current in vitro systems, such as 2D neuronal cultures, fail to recapitulate the complex multicellular interactions of the human brain. Similarly, rodent models only partially model human neurodegenerative pathology and pharmacokinetics. Differences in BBB permeability, lifespan, and cognitive complexity impede assessment of subtle or delayed neurotoxic or neuroprotective effects of chronic PCSK9 inhibition, reducing translational relevance.

Moreover, a critical limitation of the current evidence base is the relative scarcity of studies specifically designed to evaluate PCSK9 inhibitors in neurodegeneration-relevant contexts. While preclinical studies have examined PCSK9 modulation in AD (5 × FAD mice), stroke, and alcohol-induced neurotoxicity (Fig. 3), there is a striking absence of data from classic PD models, including toxin (MPTP, 6-OHDA)-based models and α-synuclein PFF models. Similarly, no studies have investigated PCSK9 inhibition in tauopathy models (e.g., P301S mice) or in transgenic (*SOD1*, *C9orf72*, *TDP-43*) models of ALS. This gap limits our understanding of whether the neuroprotective effects observed in AD and stroke models can generalize to other neurodegenerative conditions with distinct proteinopathies and cell-type vulnerabilities.

On the clinical side, although large cardiovascular outcome trials have provided reassuring cognitive safety data, none were designed with neurodegenerative disease progression as a primary endpoint. Patients with established mild cognitive impairment, AD, PD, or other neurodegenerative conditions have been systematically excluded from these trials. Consequently, we lack direct evidence regarding whether PCSK9 inhibitors modify disease trajectories in affected individuals. Furthermore, no trials have incorporated neurodegeneration-specific biomarkers (e.g., plasma phosphorylated tau, neurofilament light chain, dopamine transporter imaging) as outcome measures, limiting mechanistic inference from human studies.

### Incomplete mechanistic resolution

Additional uncertainty arises from the incomplete definition of mechanistic links between systemic lipid metabolism, circulating PCSK9 levels, and CNS effects. While BBB integrity typically restricts peripheral PCSK9 entry into the CNS, the states of inflammation or hyperlipidemia may increase permeability, creating potential feedback loops that are yet to be fully characterized. Concurrent use of statins and other lipid-lowering agents in most study populations complicates attribution of observed effects to PCSK9 inhibition alone. A key unresolved question is the relative contribution of direct CNS effects versus indirect vascular-mediated effects of PCSK9 inhibition to any observed neurocognitive outcomes.

### Challenges from confounding effects in real-world observational data

Real-world observational studies attempt to address some of these gaps but introduce their own challenges, including confounding by indication, variability in medication adherence, and incomplete capture of cognitive outcomes in routine practice. Although pharmacovigilance analyses suggest a low overall rate of reported neuropsychiatric adverse events, underreporting or misclassification cannot be excluded, limiting their reliability.

### Methodological limits of genetic and epidemiologic inference

Genetic and epidemiologic approaches add another layer of complexity. Mendelian randomization analyses and genetic association studies provide conflicting results regarding the role of PCSK9 in neurodegenerative risk. These methods are limited by pleiotropy, population stratification, and potential residual confounding.

### Heterogeneity of neurodegenerative disorders and underpowered subgroup assessment

Finally, the heterogeneity of neurodegenerative diseases, including AD, vascular dementia, PD, and ALS, implies that PCSK9 inhibition may exert divergent effects across disorders, stages, and patient subgroups. Existing studies are underpowered to detect these nuanced or subgroup-specific effects, highlighting the need for larger, stratified trials with disease-specific endpoints.

Collectively, these limitations call for (1) enriched/longer clinical trials with sensitive cognitive, biomarker and imaging end points, (2) human-relevant mechanistic studies addressing specific LDLR-family receptors and BBB dynamics, and (3) robust real-world surveillance and genetic triangulation. The recommendations below outline concrete, feasible study designs to address each priority.

### Limitations of current trials

While completed clinical trials consistently indicate no significant cognitive harm from PCSK9 inhibitors, several methodological limitations must be recognized. First, the follow-up durations in published trials have been relatively short – typically about 2 to 3 years – which may be insufficient to detect subtle or delayed cognitive changes.

Second, the trial populations have generally been middle-aged or “younger-old” adults at cardiovascular risk, rather than very elderly individuals at highest risk for neurodegeneration. For example, in the FOURIER and ODYSSEY Outcomes trials, the median ages were about 62 and 58 years, respectively, with only a minority of patients being over 75. Moreover, patients with baseline cognitive impairment or recent strokes were often excluded, yielding cohorts that were at a low risk for cognitive decline at baseline. This “healthy cohort” effect (including a possible volunteer bias among those who completed cognitive testing) could mask adverse effects that might emerge in more vulnerable populations.

Third, the sensitivity of the cognitive assessment tools used in these trials is a concern. Objective test batteries like the Cambridge Neuropsychological Test Automated Battery (CANTAB, used in EBBINGHAUS) were administered over the short trial period and may not capture very subtle declines in complex cognitive abilities. Conversely, simpler instruments such as the ECog questionnaire (used as a one-time patient survey in FOURIER’s cohort) rely on self-reports and were only assessed at study end. Future studies with extended follow-up, older and more cognitively at-risk participants, and more sensitive, multidomain cognitive testing (potentially alongside biomarkers) will be crucial to fully address these limitations.

Lastly, nearly all participants in the major PCSK9 inhibitor trials were on concurrent statin therapy. Statins have a controversial but largely weak link to cognitive changes – with post-marketing reports of reversible memory loss, contrasted by other data suggesting that statins might protect against dementia via stroke risk reduction [108]. Thus, without a statin-free control group, current trials cannot fully rule out a modest statin impact or synergistic risk, though no clear interaction has been reported to date.

### Conflicting results from genetic and Mendelian randomization studies

Conflicting findings regarding AD/PD risk with PCSK9 inhibition often stem from differences in study design and data sources. Some analyses (e.g. large meta-analyses using IGAP Alzheimer’s cohorts) found that genetically proxied PCSK9 inhibition is associated with increased AD risk [109], whereas others (e.g. single-cohort or broad lipid variant studies) reported no effect or even protective trends [110]. For example, one Mendelian randomization study using multiple PCSK9 locus variants (weighted by LDL reduction) predicted a higher odds of AD (OR ~ 1.45 per SD lower LDL-C) [109]. In contrast, an earlier Mendelian randomization analysis combining *PCSK9* and *HMGCR* loss-of-function variants found no significant causal effect on AD or PD risk – in fact, life-long LDL lowering by any genetic means was associated with a lower AD risk overall (OR ~ 0.64) [110]. These discrepancies highlight that results can vary based on instrument selection & statistical power, where studies using a broad panel of LDL-lowering variants (hundreds of SNPs) tended to have greater power and showed a robust protective effect on AD. In contrast, analyses focusing narrowly on PCSK9 may rely on one or few variants (e.g. *PCSK9* R46L or gene scores) – these can yield different estimates with wider confidence intervals or opposite direction due to lower power or single-gene influences. Furthermore, data sources and sample differences may have also contributed to differences in results. Fo example, results from a single population-based cohort (e.g. Danish general population in Benn et al. 2017) [110] might differ from international consortia meta-analyses (e.g. IGAP for AD, or large GWAS for PD) [111]. Population characteristics (age, environmental factors) and case definitions can vary, influencing outcomes. For instance, while the Danish study found no genetic link to PD, a more recent multi-cohort Mendelian randomization study reported a modest but significant increase in PD risk with *PCSK9* variants (OR ~ 1.42). The latter drew on larger GWAS datasets, potentially detecting effects that smaller studies missed. However, cross-cohort differences (e.g. ancestry or diagnostic criteria) could also contribute to the inconsistent results. Moreover, differences in outcome ascertainment could have led to differences in risk prediction of neurodegenerative disorders. The definition of AD or PD can differ (clinical diagnosis vs. International Classification of Diseases (ICD) codes vs. research criteria) and the presence of comorbid conditions or competing risks may confound results.

In addition, pleiotropy is a key concern in genetic analyses of *PCSK9*. Mendelian randomization assumes that genetic instruments affect the outcome only through LDL lowering. In reality, however, PCSK9 variants have diverse biological effects beyond cholesterol. For instance, *PCSK9* loss-of-function alleles also lower lipoprotein (a) levels and influence glucose metabolism, with direct roles in the brain (neuronal apoptosis, inflammation, synaptic function). These off-target effects (horizontal pleiotropy) could bias the Mendelian randomization results. A genetic score might therefore associate with neurodegenerative disease risk through mechanisms unrelated to LDL cholesterol – e.g. altering amyloid clearance or neuroinflammation independently of lipid levels.

### Clinical implications and future trials in pipeline

Current evidence largely indicates that PCSK9 inhibitors do not impair cognitive function in the general patient population, but important limitations persist. Crucially, most clinical trials and cognitive studies to date have enrolled relatively low-risk cohorts (middle-aged or younger older adults with cardiovascular disease) and followed them for only ~ 2 years. For example, the EBBINGHAUS trial showed no between-group differences in cognitive performance with evolocumab over a median 19–24 months[60]. However, such trials excluded or underrepresented high-risk neurocognitive populations (e.g. elderly patients, *APOE* ε4 carriers at genetic risk for AD, or individuals with pre-existing mild cognitive impairment). In addition, cognitive outcomes were usually secondary measures or based on patient-reported complaints, introducing potential reporting bias and insensitivity to mild changes. Sample sizes and follow-up durations in published trials were not powered to detect rare events or slow, progressive cognitive decline.

Given these gaps, it remains possible that rare, subtle, or late-onset neurocognitive effects of PCSK9 inhibition could have been missed. The open-label OSLER-1 extension [112] and ODYSSEY LONG TERM trial [68] reported a higher incidence of patient-reported memory loss in those on PCSK9 inhibitor treatment compared to placebo treatment (though the absolute event numbers were low). These subjective findings did not translate into objective deficits in formal testing, yet they underscore the need for caution.

To directly address the remaining uncertainties, new clinical studies are targeting populations with elevated neurocognitive risk or pre-existing impairment. One notable ongoing trial is evaluating aggressive LDL-C lowering in patients with asymptomatic intracranial atherosclerosis – a condition linked to vascular cognitive impairment. In this Chinese trial (PISTIAS-2, PCSK9 Inhibitor with Statin Therapy for Asymptomatic Intracranial Atherosclerosis; NCT06902740), patients are randomized to the PCSK9 monoclonal antibody (recaticimab) plus high-intensity statin group versus the statin alone group [92]. Besides vascular endpoints, the investigators are tracking cognitive outcomes using volumetric analysis, given that intracranial atherosclerosis and hyperlipidemia may accelerate neurodegeneration. This study specifically enriches for a high-risk cognitive group (older adults with cerebrovascular disease, some with baseline mild cognitive impairment) and will provide prospective data on whether intensive PCSK9 inhibition influences cognitive trajectories in a vulnerable population. Additionally, ongoing longitudinal registries and genetic studies in familial hypercholesterolemia patients (who often carry lifelong extreme LDL levels and sometimes PCSK9 mutations) may shed light on the late-life cognitive outcomes with and without PCSK9 inhibitor treatment. Importantly, to date no clinical trials have been designed exclusively to test cognitive end points in *APOE4* carriers or AD patients on PCSK9 inhibitors. Nonetheless, the overlap of cardiovascular risk management and cognitive health is drawing increasing attention.

## Future directions

### Long-term clinical trials

Addressing the current limitations will require a staged and multifaceted research strategy that integrates clinical, mechanistic, and translational approaches. Long-term randomized controlled trials specifically designed to evaluate neurocognitive outcomes in older adults, *APOE4* carriers, and patients with pre-existing cognitive impairment are essential. These trials should extend follow-up beyond five years and incorporate standardized, domain-specific neuropsychological batteries, serial biomarker assessments (e.g., neurofilament light chain, phospho-tau), and advanced neuroimaging modalities (amyloid and tau PET, functional MRI) to detect subtle or early changes. The feasibility of such studies lies in embedding cognitive endpoints within ongoing cardiovascular outcome trials, despite challenges including high-cost, recruitment of high-risk populations, and patient adherence over extended periods.

### Real-world evidence and surveillance

Real-world evidence must be strengthened through large-scale, prospective registries with standardized cognitive follow-up, pharmacogenomic profiling, and robust adjustment for confounding factors. Enhanced post-marketing surveillance systems, linked to electronic health records and claims data, can help detect rare or delayed neuropsychiatric events. While feasible within existing health informatics infrastructures, challenges include harmonizing data across diverse healthcare systems and ensuring adequate capture of cognitive outcomes.

### Pathophysiologic studies

Future research should also prioritize pathophysiologic investigations to clarify the mechanistic role of PCSK9 in neurodegenerative processes. Human-derived models such as iPSC-based brain organoids and BBB-on-chip systems can be leveraged to dissect receptor-specific pathways, including LDLR, ApoER2, VLDLR, and LRP1, in synaptic plasticity, amyloid-beta clearance, and myelination. CRISPR-engineered lines carrying disease-relevant mutations (e.g., *APOE4*, tau, α-synuclein) will enable exploration of gene–drug interactions and disease-specific vulnerabilities. Complementary in vivo studies in non-human primates may provide critical insights into age-related proteinopathies, neuroinflammation, and BBB dynamics in a physiologic context closer to humans. These pathophysiologic studies are essential to bridge the gap between systemic lipid regulation and CNS-specific effects, helping to identify causal pathways and therapeutic targets that can inform precision medicine strategies. To transition from hypothesis-generating to evidence-based clinical application, dedicated trials are urgently needed. Future studies should move beyond secondary safety analyses and design trials with primary neurodegenerative endpoints. Specifically, long-term PCSK9 inhibitor trials should incorporate robust cognitive batteries, volumetric neuroimaging, and fluid biomarkers (such as CSF phospho-tau (p-tau) or plasma neurofilament light chain (NfL)) to prospectively track disease modification in high-risk groups, including *APOE4* carriers and patients with early-stage AD or PD.

### Multi-omics approaches

Multi-omics approaches, including single-cell transcriptomics, proteomics, and lipidomics, can be used to illuminate patient subgroups most likely to benefit or experience harm from PCSK9 inhibition. In particular, patients can be better stratified according to cholesterol genetic risk factors or targets for reduction. While technically feasible, challenges exist including integrating large datasets across platforms and translating molecular signatures into clinically actionable insights.

### Precision medicine frameworks

Precision medicine frameworks incorporating *APOE* genotype, lipidomic profiles, and environmental or lifestyle risk factors should be developed to tailor PCSK9 inhibitor use in populations at elevated risk for neurodegeneration. Such frameworks will require interdisciplinary collaboration between neurologists, cardiologists, geneticists, and data scientists. The feasibility of this approach is supported by the increasing availability of biobanking and genotyping resources, though challenges remain in validating predictive models across diverse populations.

## Conclusion

Current clinical evidence supports a reassuring cognitive safety profile for PCSK9 inhibitors in cardiovascular trial populations, including settings of intensive LDL-C lowering and extended follow-up. However, these studies primarily address cognitive safety rather than neurodegenerative disease modification, as most were not designed with neurodegeneration-specific endpoints, CNS target engagement, or longitudinal biomarker and imaging readouts. Although mechanistic and genetic studies suggest that PCSK9 may intersect with pathways relevant to neurodegeneration, including neuroinflammation, BBB biology, and amyloid-β handling, these findings remain hypothesis-generating. Accordingly, PCSK9 inhibitors should presently be viewed as cardiovascular therapeutics with no clear signal of cognitive harm, while any role in modifying neurodegenerative trajectories remains unproven and requires dedicated prospective studies in at-risk populations using standardized cognitive, fluid biomarker, neuroimaging, and central target-engagement measures.

## Data Availability

Not applicable.

## Abbreviations

Aβ: Amyloid-beta
AD: Alzheimer’s disease
ALS: Amyotrophic lateral sclerosis
APOE: Apolipoprotein E
BBB: Blood–brain barrier
CANTAB: Cambridge Neuropsychological Test Automated Battery
CNS: Central nervous system
CSF: Cerebrospinal fluid
CV: Cardiovascular
ECog: Everyday Cognition
FH: Familial hypercholesterolemia
GWAS: Genome-wide association study
LDL-C: Low-density lipoprotein cholesterol
LDLR: Low-density lipoprotein receptor
LRP1: Low-density lipoprotein receptor-related protein 1
MoCA: Montreal Cognitive Assessment
PCSK9: Proprotein convertase subtilisin/kexin type 9
PD: Parkinson’s disease
PNS: Peripheral nervous system
RCT: Randomized controlled trial
ROS: Reactive oxygen species
siRNA: Small interfering RNA
SWMS: Spatial working memory strategy
TLR: Toll-like receptor
VLDLR: Very low-density lipoprotein receptor

## References

[1] Abifadel M, Varret M, Rabes JP, Allard D, Ouguerram K, Devillers M, et al. Mutations in PCSK9 cause autosomal dominant hypercholesterolemia. Nat Genet. 2003;34(2):154–6. 12730697 10.1038/ng1161

[2] Ference BA, Ginsberg HN, Graham I, Ray KK, Packard CJ, Bruckert E, et al. Low-density lipoproteins cause atherosclerotic cardiovascular disease. Evidence from genetic, epidemiologic, and clinical studies. A consensus statement from the European Atherosclerosis Society Consensus Panel. Eur Heart J. 2017;38(32):2459–72. 28444290 10.1093/eurheartj/ehx144 PMC5837225

[3] Lepor NE, Kereiakes DJ. The PCSK9 inhibitors: a novel therapeutic target enters clinical practice. Am Health Drug Benefits. 2015;8(9):483–9. 26834934 PMC4719137

[4] Sabatine MS, Giugliano RP, Keech AC, Honarpour N, Wiviott SD, Murphy SA, et al. Evolocumab and clinical outcomes in patients with cardiovascular disease. N Engl J Med. 2017;376(18):1713–22. 28304224 10.1056/NEJMoa1615664

[5] Ray KK, Wright RS, Kallend D, Koenig W, Leiter LA, Raal FJ, et al. Two phase 3 trials of inclisiran in patients with elevated LDL cholesterol. N Engl J Med. 2020;382(16):1507–19. 32187462 10.1056/NEJMoa1912387

[6] Poirier S, Mayer G, Benjannet S, Bergeron E, Marcinkiewicz J, Nassoury N, et al. The proprotein convertase PCSK9 induces the degradation of low density lipoprotein receptor (LDLR) and its closest family members VLDLR and ApoER2. J Biol Chem. 2008;283(4):2363–72. 18039658 10.1074/jbc.M708098200

[7] Vilella A, Bodria M, Papotti B, Zanotti I, Zimetti F, Remaggi G, et al. PCSK9 ablation attenuates Abeta pathology, neuroinflammation and cognitive dysfunctions in 5XFAD mice. Brain Behav Immun. 2024;115:517–34. 37967665 10.1016/j.bbi.2023.11.008

[8] O’Connell EM, Lohoff FW. Proprotein convertase subtilisin/kexin type 9 (PCSK9) in the brain and relevance for neuropsychiatric disorders. Front Neurosci. 2020;14:609. 32595449 10.3389/fnins.2020.00609 PMC7303295

[9] Schwartzentruber J, Cooper S, Liu JZ, Barrio-Hernandez I, Bello E, Kumasaka N, et al. Genome-wide meta-analysis, fine-mapping and integrative prioritization implicate new Alzheimer’s disease risk genes. Nat Genet. 2021;53(3):392–402. 33589840 10.1038/s41588-020-00776-w PMC7610386

[10] Picard C, Poirier A, Belanger S, Labonte A, Auld D, Poirier J. Proprotein convertase subtilisin/kexin type 9 (PCSK9) in Alzheimer’s disease: a genetic and proteomic multi-cohort study. PLoS One. 2019;14(8):e0220254. 31437157 10.1371/journal.pone.0220254 PMC6705826

[11] Mariani E, Frabetti F, Tarozzi A, Pelleri MC, Pizzetti F, Casadei R. Meta-analysis of Parkinson’s disease transcriptome data using TRAM software: whole substantia nigra tissue and single dopamine neuron differential gene expression. PLoS ONE. 2016;11(9):e0161567. 27611585 10.1371/journal.pone.0161567 PMC5017670

[12] Bai XQ, Peng J, Wang MM, Xiao J, Xiang Q, Ren Z, et al. PCSK9: A potential regulator of apoE/apoER2 against inflammation in atherosclerosis? Clin Chim Acta. 2018;483:192–6. 29727700 10.1016/j.cca.2018.04.040

[13] Adorni MP, Ruscica M, Ferri N, Bernini F, Zimetti F. Proprotein convertase subtilisin/kexin type 9, brain cholesterol homeostasis and potential implication for Alzheimer’s disease. Front Aging Neurosci. 2019;11:120. 31178716 10.3389/fnagi.2019.00120 PMC6538876

[14] Park LM, Pacher P, Lohoff FW. Targeting oxidative stress in neurodegenerative disorders: a novel role for PCSK9 inhibition? ACS Chem Neurosci. 2024;15(15):2662–4. 39022840 10.1021/acschemneuro.4c00299

[15] Wagner J, Park LM, Mukhopadhyay P, Matyas C, Trojnar E, Damadzic R, et al. PCSK9 inhibition attenuates alcohol-associated neuronal oxidative stress and cellular injury. Brain Behav Immun. 2024;119:494–506. 38657842 10.1016/j.bbi.2024.04.022

[16] Lagace TA, Curtis DE, Garuti R, McNutt MC, Park SW, Prather HB, et al. Secreted PCSK9 decreases the number of LDL receptors in hepatocytes and in livers of parabiotic mice. J Clin Invest. 2006;116(11):2995–3005. 17080197 10.1172/JCI29383 PMC1626117

[17] Gao Y, Ye S, Tang Y, Tong W, Sun S. Brain cholesterol homeostasis and its association with neurodegenerative diseases. Neurochem Int. 2023;171:105635. 37949118 10.1016/j.neuint.2023.105635

[18] Ray KK, Landmesser U, Leiter LA, Kallend D, Dufour R, Karakas M, et al. Inclisiran in patients at high cardiovascular risk with elevated LDL cholesterol. N Engl J Med. 2017;376(15):1430–40. 28306389 10.1056/NEJMoa1615758

[19] Blom DJ, Dent R, Castro RC, Toth PP. PCSK9 inhibition in the management of hyperlipidemia: focus on evolocumab. Vasc Health Risk Manag. 2016;12:185. 27274264 10.2147/VHRM.S102564 PMC4868869

[20] Schmidt AF, Carter JL, Pearce LS, Wilkins JT, Overington JP, Hingorani AD, et al. PCSK9 monoclonal antibodies for the primary and secondary prevention of cardiovascular disease. Cochrane Database Syst Rev. 2017;10(10):CD011748. 10.1002/14651858.CD011748.pub3 PMC8094613 33078867

[21] Musunuru K, Chadwick AC, Mizoguchi T, Garcia SP, DeNizio JE, Reiss CW, et al. In vivo CRISPR base editing of PCSK9 durably lowers cholesterol in primates. Nature. 2021;593(7859):429–34. 34012082 10.1038/s41586-021-03534-y

[22] Tavori H, Giunzioni I, Fazio S. PCSK9 inhibition to reduce cardiovascular disease risk: recent findings from the biology of PCSK9. Curr Opin Endocrinol Diabetes Obes. 2015;22(2):126–32. 25692926 10.1097/MED.0000000000000137 PMC4384821

[23] Rousselet E, Marcinkiewicz J, Kriz J, Zhou A, Hatten ME, Prat A, et al. PCSK9 reduces the protein levels of the LDL receptor in mouse brain during development and after ischemic stroke. J Lipid Res. 2011;52(7):1383–91. 21518694 10.1194/jlr.M014118 PMC3111744

[24] Zhang X, Xu H, Yu J, Cui J, Chen Z, Li Y, et al. Immune regulation of the liver through the PCSK9/CD36 pathway during heart transplant rejection. Circulation. 2023. 10.1161/CIRCULATIONAHA.123.062788. 37232170 10.1161/CIRCULATIONAHA.123.062788

[25] Storck SE, Meister S, Nahrath J, Meißner JN, Schubert N, Di Spiezio A, et al. Endothelial LRP1 transports amyloid-β1–42 across the blood-brain barrier. J Clin Invest. 2016;126(1):123–36. 26619118 10.1172/JCI81108 PMC4701557

[26] Galea I. The blood–brain barrier in systemic infection and inflammation. Cell Mol Immunol. 2021;18(11):2489–501. 34594000 10.1038/s41423-021-00757-x PMC8481764

[27] Guan Y, Liu X, Yang Z, Zhu X, Liu M, Du M, et al. PCSK9 promotes LDLR degradation by preventing SNX17-mediated LDLR recycling. Circulation. 2025. 10.1161/circulationaha.124.072336. 40071387 10.1161/CIRCULATIONAHA.124.072336

[28] Rousselet E, Marcinkiewicz J, Kriz J, Zhou A, Hatten ME, Prat A, et al. PCSK9 reduces the protein levels of the LDL receptor in mouse brain during development and after ischemic stroke. J Lipid Res. 2011;52(7):1383–91. 21518694 10.1194/jlr.M014118 PMC3111744

[29] Parn A, Olsen D, Tuvikene J, Kaas M, Borisova E, Bilgin M, et al. PCSK9 deficiency alters brain lipid composition without affecting brain development and function. Front Mol Neurosci. 2023;15:1084633. 36733269 10.3389/fnmol.2022.1084633 PMC9887304

[30] Papotti B, Adorni MP, Marchi C, Zimetti F, Ronda N, Panighel G, et al. PCSK9 affects astrocyte cholesterol metabolism and reduces neuron cholesterol supplying in vitro: potential implications in Alzheimer’s disease. Int J Mol Sci. 2022. 10.3390/ijms232012192. 36293049 10.3390/ijms232012192 PMC9602670

[31] Saher G, Quintes S, Nave KA. Cholesterol: a novel regulatory role in myelin formation. Neuroscientist. 2011;17(1):79–93. 21343408 10.1177/1073858410373835

[32] Saher G, Stumpf SK. Cholesterol in myelin biogenesis and hypomyelinating disorders. Biochim Biophys Acta. 2015;1851(8):1083–94. 25724171 10.1016/j.bbalip.2015.02.010

[33] Orth M, Bellosta S. Cholesterol: its regulation and role in central nervous system disorders. Cholesterol. 2012;2012(1):292598. 23119149 10.1155/2012/292598 PMC3483652

[34] Barber CN, Raben DM. Lipid metabolism crosstalk in the brain: glia and neurons. Front Cell Neurosci. 2019;13:212. 31164804 10.3389/fncel.2019.00212 PMC6536584

[35] Beard E, Lengacher S, Dias S, Magistretti PJ, Finsterwald C. Astrocytes as key regulators of brain energy metabolism: new therapeutic perspectives. Front Physiol. 2022;12:2021. 10.3389/fphys.2021.825816 PMC8787066 35087428

[36] Passarella D, Ronci M, Di Liberto V, Zuccarini M, Mudò G, Porcile C, et al. Bidirectional control between cholesterol shuttle and purine signal at the central nervous system. Int J Mol Sci. 2022;23(15):8683. 35955821 10.3390/ijms23158683 PMC9369131

[37] Raefsky SM, Mattson MP. Adaptive responses of neuronal mitochondria to bioenergetic challenges: Roles in neuroplasticity and disease resistance. Free Radic Biol Med. 2017;102:203–16. 27908782 10.1016/j.freeradbiomed.2016.11.045 PMC5209274

[38] Mauch DH, Nägler K, Schumacher S, Göritz C, Müller E-C, Otto A, et al. CNS synaptogenesis promoted by glia-derived cholesterol. Science. 2001;294(5545):1354–7. 11701931 10.1126/science.294.5545.1354

[39] Jaafar AK, Techer R, Chemello K, Lambert G, Bourane S. PCSK9 and the nervous system: a no-brainer? J Lipid Res. 2023;64(9):100426. 37586604 10.1016/j.jlr.2023.100426 PMC10491654

[40] Pelucchi S, Da Dalt L, De Cesare G, Stringhi R, D’Andrea L, La Greca F, et al. Neuronal PCSK9 regulates cognitive performances via the modulation of ApoER2 synaptic localization. Pharmacol Res. 2025;213:107652. 39952371 10.1016/j.phrs.2025.107652

[41] Beffert U, Weeber EJ, Durudas A, Qiu S, Masiulis I, Sweatt JD, et al. Modulation of synaptic plasticity and memory by reelin involves differential splicing of the lipoprotein receptor Apoer2. Neuron. 2005;47(4):567–79. 16102539 10.1016/j.neuron.2005.07.007

[42] Kulik YD, Watson DJ, Cao G, Kuwajima M, Harris KM. Structural plasticity of dendritic secretory compartments during LTP-induced synaptogenesis. eLife. 2019;8:e46356. 31433297 10.7554/eLife.46356 PMC6728136

[43] Mazura AD, Ohler A, Storck SE, Kurtyka M, Scharfenberg F, Weggen S, et al. PCSK9 acts as a key regulator of Abeta clearance across the blood-brain barrier. Cell Mol Life Sci. 2022;79(4):212. 35344086 10.1007/s00018-022-04237-x PMC8960591

[44] Canuel M, Sun X, Asselin M-C, Paramithiotis E, Prat A, Seidah NG. Proprotein convertase subtilisin/kexin type 9 (PCSK9) can mediate degradation of the low density lipoprotein receptor-related protein 1 (LRP-1). PLoS ONE. 2013;8(5):e64145. 23675525 10.1371/journal.pone.0064145 PMC3652815

[45] Testa G, Giannelli S, Staurenghi E, Cecci R, Floro L, Gamba P, et al. The emerging role of PCSK9 in the pathogenesis of Alzheimer’s disease: a possible target for the disease treatment. Int J Mol Sci. 2024. 10.3390/ijms252413637. 39769398 10.3390/ijms252413637 PMC11727734

[46] Bamberger ME, Harris ME, McDonald DR, Husemann J, Landreth GE. A cell surface receptor complex for fibrillar beta-amyloid mediates microglial activation. J Neurosci. 2003;23(7):2665–74. 12684452 10.1523/JNEUROSCI.23-07-02665.2003 PMC6742111

[47] Bao X, Liang Y, Chang H, Cai T, Feng B, Gordon K, et al. Targeting proprotein convertase subtilisin/kexin type 9 (PCSK9): from bench to bedside. Signal Transduct Target Ther. 2024;9(1):13. 38185721 10.1038/s41392-023-01690-3 PMC10772138

[48] Tang ZH, Peng J, Ren Z, Yang J, Li TT, Li TH, et al. New role of PCSK9 in atherosclerotic inflammation promotion involving the TLR4/NF-kappaB pathway. Atherosclerosis. 2017;262:113–22. 28535426 10.1016/j.atherosclerosis.2017.04.023

[49] Wang F, Li M, Zhang A, Li H, Jiang C, Guo J. PCSK9 modulates macrophage polarization-mediated ventricular remodeling after myocardial infarction. J Immunol Res. 2022;2022:7685796. 35832650 10.1155/2022/7685796 PMC9273409

[50] Ricci C, Ruscica M, Camera M, Rossetti L, Macchi C, Colciago A, et al. PCSK9 induces a pro-inflammatory response in macrophages. Sci Rep. 2018;8(1):2267. 29396513 10.1038/s41598-018-20425-x PMC5797178

[51] Ling P, Zheng X, Luo S, Ge J, Xu S, Weng J. Targeting angiopoietin-like 3 in atherosclerosis: From bench to bedside. Diabetes Obes Metab. 2021;23(9):2020–34. 34047441 10.1111/dom.14450

[52] Bell AS, Wagner J, Rosoff DB, Lohoff FW. Proprotein convertase subtilisin/kexin type 9 (PCSK9) in the central nervous system. Neurosci Biobehav Rev. 2023;149:105155. 37019248 10.1016/j.neubiorev.2023.105155

[53] Scalise V, Sanguinetti C, Neri T, Cianchetti S, Lai M, Carnicelli V, et al. PCSK9 induces tissue factor expression by activation of TLR4/NFkB signaling. Int J Mol Sci. 2021. 10.3390/ijms222312640. 34884442 10.3390/ijms222312640 PMC8657476

[54] Tang Z, Jiang L, Peng J, Ren Z, Wei D, Wu C, et al. PCSK9 siRNA suppresses the inflammatory response induced by oxLDL through inhibition of NF-kappaB activation in THP-1-derived macrophages. Int J Mol Med. 2012;30(4):931–8. 22825241 10.3892/ijmm.2012.1072

[55] Pingale TD, Gupta GL. Novel therapeutic approaches for Parkinson’s disease by targeting brain cholesterol homeostasis. J Pharm Pharmacol. 2021;73(7):862–73. 33822122 10.1093/jpp/rgaa063

[56] Nozue T. Lipid lowering therapy and circulating PCSK9 concentration. J Atheroscler Thromb. 2017;24(9):895–907. 28804094 10.5551/jat.RV17012 PMC5587514

[57] Alsehli AM, Olivo G, Clemensson LE, Williams MJ, Schiöth HB. The cognitive effects of statins are modified by age. Sci Rep. 2020;10(1):6187. 32277109 10.1038/s41598-020-63035-2 PMC7148321

[58] Gentreau M, Rukh G, Miguet M, Clemensson LE, Alsehli AM, Titova OE, et al. The effects of statins on cognitive performance are mediated by low-density lipoprotein, C-reactive protein, and blood glucose concentrations. J Gerontol A Biol Sci Med Sci. 2023;78(11):1964–72. 37431946 10.1093/gerona/glad163 PMC10613010

[59] Swiger KJ, Martin SS. PCSK9 inhibitors and neurocognitive adverse events: exploring the FDA directive and a proposal for N-of-1 trials. Drug Saf. 2015;38(6):519–26. 25989944 10.1007/s40264-015-0296-6

[60] Giugliano RP, Mach F, Zavitz K, Kurtz C, Im K, Kanevsky E, et al. Cognitive function in a randomized trial of evolocumab. N Engl J Med. 2017;377(7):633–43. 28813214 10.1056/NEJMoa1701131

[61] Zimerman A, O’Donoghue ML, Ran X, Im K, Ott BR, Mach F, et al. Long-term cognitive safety of achieving very low LDL cholesterol with evolocumab. NEJM Evid. 2025;4(1):EVIDoa2400112. 39718423 10.1056/EVIDoa2400112

[62] Gencer B, Mach F, Guo J, Im K, Ruzza A, Wang H, et al. Cognition After Lowering LDL-Cholesterol With Evolocumab. J Am Coll Cardiol. 2020;75(18):2283–93. 32381158 10.1016/j.jacc.2020.03.039

[63] Seijas-Amigo J, Mauriz-Montero MJ, Suarez-Artime P, Gayoso-Rey M, Estany-Gestal A, Casas-Martínez A, et al. Cognitive function with PCSK9 inhibitors: a 24-month follow-up observational prospective study in the real world-MEMOGAL study. Am J Cardiovasc Drugs. 2023;23(5):583–93. 37612529 10.1007/s40256-023-00604-6 PMC10462529

[64] Janik MJ, Urbach DV, van Nieuwenhuizen E, Zhao J, Yellin O, Baccara-Dinet MT, et al. Alirocumab treatment and neurocognitive function according to the CANTAB scale in patients at increased cardiovascular risk: a prospective, randomized, placebo-controlled study. Atherosclerosis. 2021;331:20–7. 34303265 10.1016/j.atherosclerosis.2021.06.913

[65] Korthauer LE, Giugliano RP, Guo J, Sabatine MS, Sever P, Keech A, et al. No association between APOE genotype and lipid lowering with cognitive function in a randomized controlled trial of evolocumab. PLoS ONE. 2022;17(4):e0266615. 35404972 10.1371/journal.pone.0266615 PMC9000128

[66] Roth EM, Moriarty PM, Bergeron J, Langslet G, Manvelian G, Zhao J, et al. A phase III randomized trial evaluating alirocumab 300 mg every 4 weeks as monotherapy or add-on to statin: ODYSSEY CHOICE I. Atherosclerosis. 2016;254:254–62. 27639753 10.1016/j.atherosclerosis.2016.08.043

[67] Koren MJ, Lundqvist P, Bolognese M, Neutel JM, Monsalvo ML, Yang J, et al. Anti-PCSK9 monotherapy for hypercholesterolemia: the MENDEL-2 randomized, controlled phase III clinical trial of evolocumab. J Am Coll Cardiol. 2014;63(23):2531–40. 24691094 10.1016/j.jacc.2014.03.018

[68] Robinson JG, Farnier M, Krempf M, Bergeron J, Luc G, Averna M, et al. Efficacy and safety of alirocumab in reducing lipids and cardiovascular events. N Engl J Med. 2015;372(16):1489–99. 25773378 10.1056/NEJMoa1501031

[69] Sabatine MS, Giugliano RP, Wiviott SD, Raal FJ, Blom DJ, Robinson J, et al. Efficacy and safety of evolocumab in reducing lipids and cardiovascular events. N Engl J Med. 2015;372(16):1500–9. 25773607 10.1056/NEJMoa1500858

[70] O’Donoghue ML, Giugliano RP, Wiviott SD, Atar D, Keech A, Kuder JF, et al. Long-term evolocumab in patients with established atherosclerotic cardiovascular disease. Circulation. 2022;146(15):1109–19. 36031810 10.1161/CIRCULATIONAHA.122.061620

[71] Schwartz GG, Steg PG, Szarek M, Bhatt DL, Bittner VA, Diaz R, et al. Alirocumab and cardiovascular outcomes after acute coronary syndrome. N Engl J Med. 2018;379(22):2097–107. 30403574 10.1056/NEJMoa1801174

[72] Cannon CP, Cariou B, Blom D, McKenney JM, Lorenzato C, Pordy R, et al. Efficacy and safety of alirocumab in high cardiovascular risk patients with inadequately controlled hypercholesterolaemia on maximally tolerated doses of statins: the ODYSSEY COMBO II randomized controlled trial. Eur Heart J. 2015;36(19):1186–94. 25687353 10.1093/eurheartj/ehv028 PMC4430683

[73] Moriarty PM, Thompson PD, Cannon CP, Guyton JR, Bergeron J, Zieve FJ, et al. Efficacy and safety of alirocumab vs ezetimibe in statin-intolerant patients, with a statin rechallenge arm: the ODYSSEY ALTERNATIVE randomized trial. J Clin Lipidol. 2015;9(6):758–69. 26687696 10.1016/j.jacl.2015.08.006

[74] Stein EA, Gipe D, Bergeron J, Gaudet D, Weiss R, Dufour R, et al. Effect of a monoclonal antibody to PCSK9, REGN727/SAR236553, to reduce low-density lipoprotein cholesterol in patients with heterozygous familial hypercholesterolaemia on stable statin dose with or without ezetimibe therapy: a phase 2 randomised controlled trial. Lancet. 2012;380(9836):29–36. 22633824 10.1016/S0140-6736(12)60771-5

[75] Teramoto T, Kobayashi M, Tasaki H, Yagyu H, Higashikata T, Takagi Y, et al. Efficacy and safety of alirocumab in Japanese patients with heterozygous familial hypercholesterolemia or at high cardiovascular risk with hypercholesterolemia not adequately controlled with statins - ODYSSEY JAPAN randomized controlled trial. Circ J. 2016;80(9):1980–7. 27452202 10.1253/circj.CJ-16-0387

[76] Blom DJ, Hala T, Bolognese M, Lillestol MJ, Toth PD, Burgess L. A 52-week placebo-controlled trial of evolocumab in hyperlipidemia. N Engl J Med. 2014;370(19):1809–19. 24678979 10.1056/NEJMoa1316222

[77] Harvey PD, Sabbagh MN, Harrison JE, Ginsberg HN, Chapman MJ, Manvelian G, et al. No evidence of neurocognitive adverse events associated with alirocumab treatment in 3340 patients from 14 randomized Phase 2 and 3 controlled trials: a meta-analysis of individual patient data. Eur Heart J. 2018;39(5):374–81. 29186504 10.1093/eurheartj/ehx661 PMC5837381

[78] Khan AR, Bavishi C, Riaz H, Farid TA, Khan S, Atlas M, et al. Increased risk of adverse neurocognitive outcomes with proprotein convertase subtilisin-kexin type 9 inhibitors. Circ Cardiovasc Qual Outcomes. 2017. 10.1161/CIRCOUTCOMES.116.003153. 28073851 10.1161/CIRCOUTCOMES.116.003153

[79] Papotti B, Palumbo M, Adorni MP, Elviri L, Chiari A, Tondelli M, et al. Influence of APOE4 genotype on PCSK9-lipids association in cerebrospinal fluid and serum of patients in the Alzheimer’s disease continuum. J Alzheimers Dis. 2024;102(1):162–72. 39497318 10.1177/13872877241284213

[80] Zimetti F, Caffarra P, Ronda N, Favari E, Adorni MP, Zanotti I, et al. Increased PCSK9 cerebrospinal fluid concentrations in Alzheimer’s disease. J Alzheimers Dis. 2016;55(1):315–20. 10.3233/JAD-160411 27662294

[81] Zimetti F, Caffarra P, Ronda N, Favari E, Adorni MP, Zanotti I, et al. Increased PCSK9 cerebrospinal fluid concentrations in Alzheimer’s disease. J Alzheimers Dis. 2017;55(1):315–20. 27662294 10.3233/JAD-160411

[82] Picard C, Poirier A, Belanger S, Labonte A, Auld D, Poirier J, et al. Proprotein convertase subtilisin/kexin type 9 (PCSK9) in Alzheimer’s disease: A genetic and proteomic multi-cohort study. PLoS ONE. 2019;14(8):e0220254. 31437157 10.1371/journal.pone.0220254 PMC6705826

[83] Courtemanche H, Bigot E, Pichelin M, Guyomarch B, Boutoleau-Bretonnière C, Le May C, et al. PCSK9 concentrations in cerebrospinal fluid are not specifically increased in Alzheimer’s disease. J Alzheimers Dis. 2018;62(4):1519–25. 29562508 10.3233/JAD-170993

[84] Williams DM, Bandres‐Ciga S, Heilbron K, Hinds D, Noyce AJ, Team aR, et al. Evaluating lipid‐lowering drug targets for Parkinson’s disease prevention with mendelian randomization. Ann Neurol. 2020;88(5):1043–7. 32841444 10.1002/ana.25880 PMC7693098

[85] O’Brien JT, Thomas A. Vascular dementia. The Lancet. 2015;386(10004):1698–706. 10.1016/S0140-6736(15)00463-8 26595643

[86] Bir SC, Khan MW, Javalkar V, Toledo EG, Kelley RE. Emerging concepts in vascular dementia: a review. J Stroke Cerebrovasc Dis. 2021;30(8):105864. 34062312 10.1016/j.jstrokecerebrovasdis.2021.105864

[87] Goldstein LB, Toth PP, Dearborn-Tomazos JL, Giugliano RP, Hirsh BJ, Peña JM, et al. Aggressive LDL-C lowering and the brain: impact on risk for dementia and hemorrhagic stroke: a scientific statement from the American Heart Association. Arterioscler Thromb Vasc Biol. 2023;43(10):e404-42. 37706297 10.1161/ATV.0000000000000164

[88] Iwagami M, Qizilbash N, Gregson J, Douglas I, Johnson M, Pearce N, et al. Blood cholesterol and risk of dementia in more than 1.8 million people over two decades: a retrospective cohort study. Lancet Healthy Longev. 2021;2(8):e498-506. 36097999 10.1016/S2666-7568(21)00150-1

[89] Benn M, Nordestgaard BG, Frikke-Schmidt R, Tybjærg-Hansen A. Low LDL cholesterol, PCSK9 and HMGCR genetic variation, and risk of Alzheimer’s disease and Parkinson’s disease: Mendelian randomisation study. BMJ. 2017;357:j1648. 28438747 10.1136/bmj.j1648 PMC5421439

[90] Mefford MT, Rosenson RS, Cushman M, Farkouh ME, McClure LA, Wadley VG, et al. PCSK9 variants, low-density lipoprotein cholesterol, and neurocognitive impairment. Circulation. 2018;137(12):1260–9. 29146683 10.1161/CIRCULATIONAHA.117.029785 PMC5860959

[91] Livingston G, Huntley J, Liu KY, Costafreda SG, Selbæk G, Alladi S, et al. Dementia prevention, intervention, and care: 2024 report of the Lancet standing Commission. Lancet. 2024;404(10452):572–628. 39096926 10.1016/S0140-6736(24)01296-0

[92] Hu X, Zhang Z, Liu C, Li M, Liu Y, Cheng A, et al. PCSK9 inhibitor with statin therapy for intracranial artery stenosis (PISTIAS): rationale and design of a multicenter randomized controlled trial. Int J Stroke. 2024;19(9):1071–6. 39075747 10.1177/17474930241270447

[93] Rozani V, Gurevich T, Giladi N, El-Ad B, Tsamir J, Hemo B, et al. Higher serum cholesterol and decreased Parkinson’s disease risk: a statin-free cohort study. Mov Disord. 2018;33(8):1298–305. 30145829 10.1002/mds.27413

[94] Swan AM, Thant HL, Chai CPM, Khaing HH, Thein MZ, Lei YW, et al. Clinical improvement in a patient with Parkinson’s disease and vascular dementia receiving PCSK9 inhibitor for ischemic heart disease: a case report. Search in; 2020.

[95] Huang Q, Zhang Q, Cao B. Causal relationship between PCSK9 inhibitor and common neurodegenerative diseases: A drug target Mendelian randomization study. Brain and Behavior. 2024;14(6):e3543. 38837845 10.1002/brb3.3543 PMC11151217

[96] Harnod T, Chen HJ, Li TC, Sung FC, Kao CH. A high risk of hyperlipidemia in epilepsy patients: a nationwide population-based cohort study. Ann Epidemiol. 2014;24(12):910–4. 25444891 10.1016/j.annepidem.2014.09.008

[97] Guo J, Guo J, Li J, Zhou M, Qin F, Zhang S, et al. Statin treatment reduces the risk of poststroke seizures. Neurology. 2015;85(8):701–7. 26203092 10.1212/WNL.0000000000001814

[98] Nucera B, Rinaldi F, Nardone R, Lattanzi S, Brigo F. Statins in primary prevention of poststroke seizures and epilepsy: A systematic review. Epilepsy Behav. 2020;112:107400. 32916580 10.1016/j.yebeh.2020.107400

[99] Quintana-Pájaro LJ, Ramos-Villegas Y, Cortecero-Sabalza E, Joaquim AF, Agrawal A, Narvaez-Rojas AR, et al. The effect of statins in epilepsy: a systematic review. J Neurosci Rural Pract. 2018;9(4):478–86. 30271037 10.4103/jnrp.jnrp_110_18 PMC6126295

[100] Banach M, Czuczwar SJ, Borowicz KK. Statins – Are they anticonvulsant? Pharmacol Rep. 2014;66(4):521–8. 24948050 10.1016/j.pharep.2014.02.026

[101] Huang S, Liu Y, Zhang Y, Wang Y, Gao Y, Li R, et al. Analyzing the causal relationship between lipid-lowering drug target genes and epilepsy: a Mendelian randomization study. Front Neurol. 2024. 10.3389/fneur.2024.1331537. 38523609 10.3389/fneur.2024.1331537 PMC10957583

[102] Liang Z, Zhao L, Lou Y, Liu S. Causal effects of circulating lipids and lipid-lowering drugs on the risk of epilepsy: a two-sample Mendelian randomization study. QJM. 2023;116(6):421–8. 36964718 10.1093/qjmed/hcad048

[103] Janse van Mantgem MR, Van Rheenen W, Hackeng AV, Van Es MA, Veldink JH, Van Den Berg LH, et al. Association between serum lipids and survival in patients with amyotrophic lateral sclerosis: a meta-analysis and population-based study. Neurology. 2023;100(10):e1062–71. 36460467 10.1212/WNL.0000000000201657 PMC9990853

[104] Zeng P, Zhou X. Causal effects of blood lipids on amyotrophic lateral sclerosis: a Mendelian randomization study. Hum Mol Genet. 2019;28(4):688–97. 30445611 10.1093/hmg/ddy384 PMC6360326

[105] Mariosa D, Hammar N, Malmström H, Ingre C, Jungner I, Ye W, et al. Blood biomarkers of carbohydrate, lipid, and apolipoprotein metabolisms and risk of amyotrophic lateral sclerosis: a more than 20-year follow-up of the Swedish AMORIS cohort. Ann Neurol. 2017;81(5):718–28. 28437840 10.1002/ana.24936

[106] Yan Z, Xu Y, Li K, Liu L. Association between genetically proxied lipid-lowering drug targets, lipid traits, and amyotrophic lateral sclerosis: a mendelian randomization study. Acta Neurol Belg. 2024;124(2):485–94. 37889424 10.1007/s13760-023-02393-w

[107] Li Z, Tian M, Jia H, Li X, Liu Q, Zhou X, et al. Genetic variation in targets of lipid-lowering drugs and amyotrophic lateral sclerosis risk: a Mendelian randomization study. Amyotroph Lateral Scler Frontotemporal Degener. 2024;25(1–2):197–206. 37688479 10.1080/21678421.2023.2255622

[108] Kazibwe R, Rikhi R, Mirzai S, Ashburn NP, Schaich CL, Shapiro M. Do statins affect cognitive health? a narrative review and critical analysis of the evidence. Curr Atheroscler Rep. 2024;27(1):2. 39520593 10.1007/s11883-024-01255-x PMC11550230

[109] Williams DM, Finan C, Schmidt AF, Burgess S, Hingorani AD. Lipid lowering and Alzheimer disease risk: A mendelian randomization study. Ann Neurol. 2020;87(1):30–9. 31714636 10.1002/ana.25642 PMC6944510

[110] Benn M, Nordestgaard BG, Frikke-Schmidt R, Tybjærg-Hansen A. Low LDL cholesterol, PCSK9 and HMGCR genetic variation, and risk of Alzheimer’s disease and Parkinson’s disease: Mendelian randomisation study. BMJ. 2017;357:j1648. 28438747 10.1136/bmj.j1648 PMC5421439

[111] Huang Q, Zhang Q, Cao B. Causal relationship between PCSK9 inhibitor and common neurodegenerative diseases: A drug target Mendelian randomization study. Brain Behav. 2024;14(6):e3543. 38837845 10.1002/brb3.3543 PMC11151217

[112] Koren MJ, Sabatine MS, Giugliano RP, Langslet G, Wiviott SD, Kassahun H, et al. Long-term low-density lipoprotein cholesterol-lowering efficacy, persistence, and safety of evolocumab in treatment of hypercholesterolemia: results up to 4 years from the Open-Label OSLER-1 extension study. JAMA Cardiol. 2017;2(6):598–607. 28291870 10.1001/jamacardio.2017.0747 PMC5815032

